# Genomic and metabolic analyses reveal antagonistic lanthipeptides in archaea

**DOI:** 10.1186/s40168-023-01521-1

**Published:** 2023-04-14

**Authors:** Haoyu Liang, Zhi-Man Song, Zheng Zhong, Dengwei Zhang, Wei Yang, Le Zhou, Ethan A. Older, Jie Li, Huan Wang, Zhirui Zeng, Yong-Xin Li

**Affiliations:** 1 Department of Chemistry and The Swire Institute of Marine Science, The University of Hong Kong, Pokfulam Road, Hong Kong, China; 2 The University of Hong Kong Shenzhen Institute of Research and Innovation, Shenzhen, China; 3 Chemistry and Chemical Engineering Guangdong Laboratory, Shantou, 515031 China; 4 Southern Marine Science and Engineering Guangdong Laboratory (Guangzhou), Guangzhou, China; 5 Department of Ocean Science and Engineering, Southern University of Science and Technology, Shenzhen, 518055 China; 6 Department of Chemistry and Biochemistry, University of South Carolina, Columbia, SC USA; 7 State Key Laboratory of Coordination Chemistry, Chemistry and Biomedicine Innovation Center of Nanjing University, School of Chemistry and Chemical Engineering, Nanjing University, Nanjing, China

## Abstract

**Background:**

Microbes produce diverse secondary metabolites (SMs) such as signaling molecules and antimicrobials that mediate microbe-microbe interaction. Archaea, the third domain of life, are a large and diverse group of microbes that not only exist in extreme environments but are abundantly distributed throughout nature. However, our understanding of archaeal SMs lags far behind our knowledge of those in bacteria and eukarya.

**Results:**

Guided by genomic and metabolic analysis of archaeal SMs, we discovered two new lanthipeptides with distinct ring topologies from a halophilic archaeon of class Haloarchaea. Of these two lanthipeptides, archalan α exhibited anti-archaeal activities against halophilic archaea, potentially mediating the archaeal antagonistic interactions in the halophilic niche. To our best knowledge, archalan α represents the first lantibiotic and the first anti-archaeal SM from the archaea domain.

**Conclusions:**

Our study investigates the biosynthetic potential of lanthipeptides in archaea, linking lanthipeptides to antagonistic interaction via genomic and metabolic analyses and bioassay. The discovery of these archaeal lanthipeptides is expected to stimulate the experimental study of poorly characterized archaeal chemical biology and highlight the potential of archaea as a new source of bioactive SMs.

Video Abstract

**Supplementary Information:**

The online version contains supplementary material available at 10.1186/s40168-023-01521-1.

## Introduction

Archaea, the third domain of life, constitute a significant fraction of the Earth’s ecosystems. Recent advances in sequencing-based approaches are revolutionizing our understanding of archaeal diversity and their metabolic and biological roles [1–5]. Distinct from bacteria and eukarya, archaea possess unique cell components and distinctive metabolic pathways. On the other hand, like bacteria and eukarya, archaea have recently been identified as an essential component in the complex microbiome, shaping their community through profound competitive or cooperative interactions [6]. Bacteria and fungi are well-known for producing diverse secondary metabolites (SMs), such as signaling molecules, antibiotics, and siderophores that mediate interactions with their biotic and abiotic environments. However, archaeal SMs remain mysterious regarding chemical structures and biosynthetic pathways, not to mention their biological functions [7–9].

Numerous antagonistic assays among archaea isolates have indicated that archaea as keystone species in microbiota are highly interactive through antagonism in competing for nutrients [5, 10–12]. For instance, Haloarchaea are well known for their antagonistic interaction toward phylogenetically related strains of halophilic archaea in the halophilic niche [5, 11, 13, 14]. However, the antagonistic origin of Haloarchaea remains unclear [5, 15, 16]. So far, only two archaeocins, the membrane-associated protein sulfolobicins from Sulfolobales and the secretory protein/peptide halocins from Haloarchaea [11–14, 17], have been described and proposed as antagonistic components in such interactions. These archaeocins were characterized solely based on the bioassay or omics analysis of antagonistic halophilic isolates. To the best of our knowledge, none of their chemical structures was fully characterized. Additionally, the contribution of archaeocins in archaeal antagonistic interactions remains elusive [5, 11, 13, 14, 16, 18–20]. Whether archaea produce SMs other than protein-based archaeocins to mediate antagonistic interactions in their competitive niche attracts our attention. With the recent explosion of sequenced archaeal genomes, a few families of biosynthetic gene clusters (BGCs) of archaeal SMs have been identified in silico, including ribosomally synthesized and post-translationally modified peptides (RiPPs) and terpene BGCs [11, 15, 21–24]. Of note, several archaeal lanthipeptide BGCs had been recently predicted via searching class II lanthipeptide hallmark protein LanM [5], suggesting the untapped genetic potential of lanthipeptides harbored in Haloarchaea. For instance, Castro et al. bioinformatically identified 40 lanthipeptide BGCs exclusively from halophilic archaea and grouped their putative LanAs into some subfamilies which shared a conserved Kx(Y/F)(D/E)xx(F/Y) motif in their leader region [5]. However, no chemical structure corresponding to these reported LanAs has been identified yet. Up to now, other than halocin [11] and membrane terpene bacterioruberin [15], no archaeal BGC has been confidently linked to secondary metabolite production. The scarcity of chemical and genetic information on archaeal SMs hinders the discovery of antagonistic metabolites and their studies on biosynthetic machinery and ecological function.

Given the metabolic potential and biological importance of archaea in the ecosystem as well as in the human microbiome [6], we sought to obtain insight into the untapped biosynthetic potential of archaeal SMs via a preliminary BGC analysis of 6412 de-replicated archaeal genomes. We envision the diverse archaeal SMs playing a vital role in their antagonistic interactions. As a proof of principle, guided by genomic and metabolic analysis, we identified two new lanthipeptides, archalans α and β. Particularly, archalan α exhibits narrow-spectrum anti-archaeal activity against closely related Haloarchaea species, potentially mediating the archaeal antagonistic interactions. To our best knowledge, this is the first report of anti-archaeal SMs from archaea. Our findings of antagonistic archalan reveal archaea as a new source of bioactive compounds and provide insight into the poorly characterized archaea-archaea interactions in microbiota.

## Results and discussion

### Biosynthetic analysis of archaeal SMs revealed untapped lanthipeptide BGCs

Intensive secondary metabolite studies have led to the accumulation of knowledge regarding natural product chemistry and biosynthetic machinery, which has greatly contributed to the development of genome mining tools such as antiSMASH [25]. In our initial analysis of archaeal biosynthetic potential, we applied antiSMASH 6.0 to 6412 selected archaeal genomes from the NCBI database. Up to 2489 genomes were found to harbor a total of 4803 antiSMASH-predicted BGCs, with the number of predicted BGCs per genome ranging from 1 to 14. Although archaea harbored a relatively low BGC number per genome compared to the more well-studied bacteria domain, they encoded most of the known classes of secondary metabolites (Supplementary Fig. 1a, b), including RiPPs, terpenes, nonribosomal peptides (NRPs), polyketides (PKs), and other metabolites. To further gain insight into the novelty of archaeal BGCs, we compared these 4803 BGCs to the reference known BGCs described in the “Minimum Information about a Biosynthetic Gene” (MIBiG) repository [26]. Only 2.4% of BGCs were found to be remotely related to characterized BGCs, leaving the vast majority completely unknown (Supplementary Fig. 1c, d). However, applying antiSMASH to detect archaeal BGCs should not be taken at face value, because some BGCs might be related to the biosynthesis of archaea-specific co-factors and SMs not related to interspecies-conflicts. Further studies are necessary to clarify the chemistry and biosynthesis of those BGCs.

**Fig. 1:**
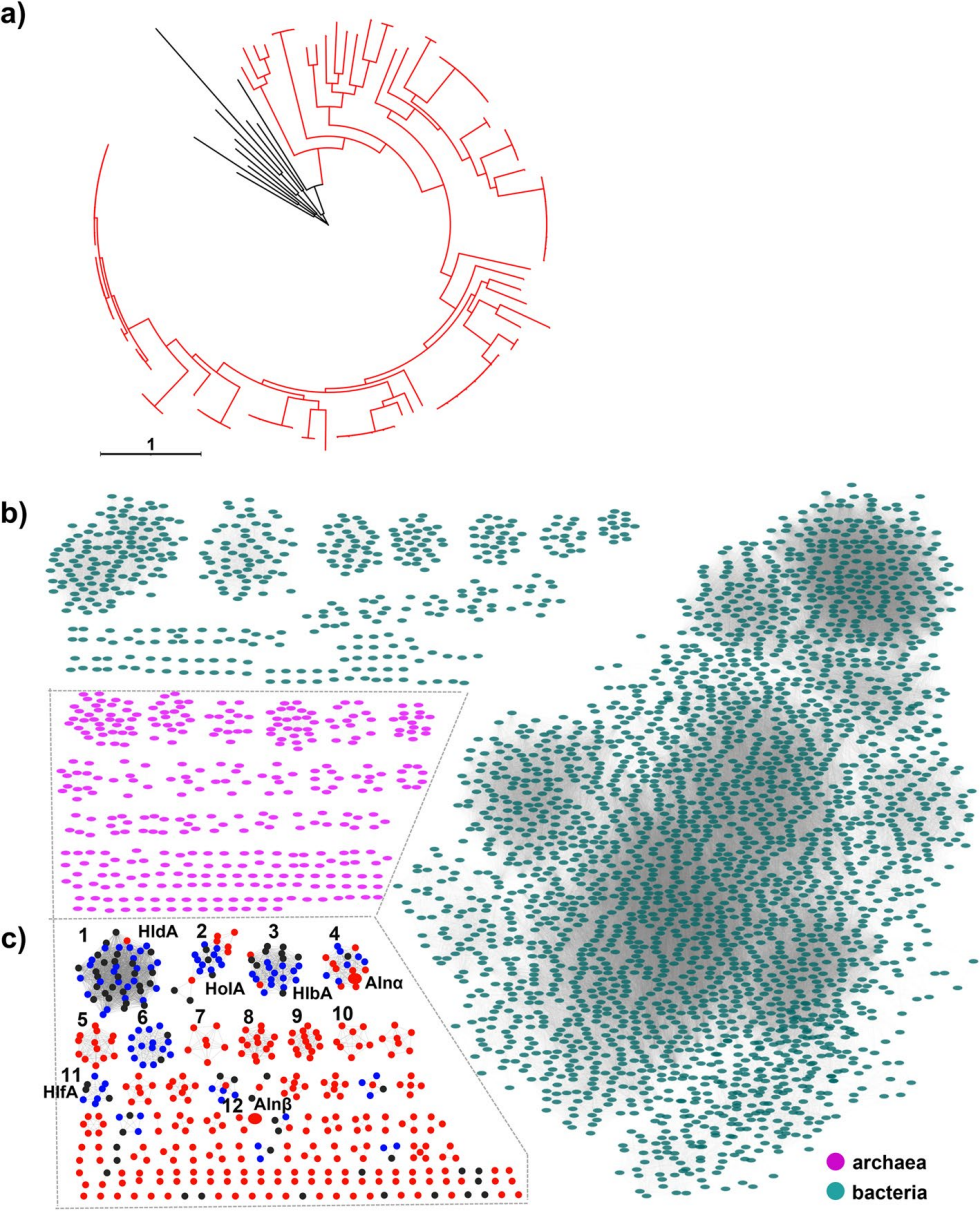
Genomic analyses reveal new lanthipeptides from archaea. **a** Phylogenetic analysis of LanMs reveals the novelty of archaeal class II lanthipeptide LanMs. The red branch represents archaeal LanMs, and the black branch represents bacterial LanMs used as the outgroups. **b** Sequence similarity network (SSN) of precursor peptides from archaea (purple) and bacteria (green). **c** The SSN of archaeal class II lanthipeptide precursors (red and blue) identified in this study and bioinformatically predicted precursors in previous studies (black). The blue and red nodes represent the precursors identical to previous studies and unique ones identified in this study, respectively. Characterized Alnα and Alnβ in this study are highlighted in big size

Nevertheless, our genomic analysis identified several BGC families with high confidence, including NRPs, PKs, NRPS-like, and well-characterized RiPP families such as lanthipeptides (Supplementary Fig. 2), highlighting archaea as an untapped source of novel chemistry. Particularly, 50 representative lanthipeptide BGCs (identified from 6412 genomes with high completeness) were mainly distributed in phylum Euryarchaeota, which is in line with the previous report [5]. Among them, 48 BGCs were classified as class II lanthipeptides harboring 50 *lanM*s (Supplementary data file). Most class II lanthipeptide BGCs contained genes encoding putative precursor peptide LanA, a typical LanM enzyme with a lanthionine synthetase C-like domain, and transporter (Supplementary Figs. 2 and 3), providing a basis for our genomic-guided discovery of lanthipeptide from archaea. Of note, a clade containing four new BGCs was far away from other clades in the phylogenetic tree (Supplementary Fig. 3), suggesting the feasibility of antiSMASH in mining novel lanthipeptide BGCs from archaea. To obtain insight into archaeal class II lanthipeptides, we applied antiSMASH to all the 9198 publicly available archaeal genomes (Supplementary data file), identifying a total of 96 class II lanthipeptide BGCs containing 103 LanMs. Our finding not only covered most of the LanMs reported in Castro et al. [5] and Walker et al. [24] except for a few truncated LanMs but also revealed 53 more new LanMs, significantly expanding the sequence space of archaeal LanMs. Phylogenetic analysis of the hallmark class II lanthipeptide synthetase LanMs showed that archaeal LanMs did not cluster with bacterial LanMs (Fig. 1a) and were closely related to Proteobacteria LanMs (Supplementary Fig. 4).

We next sought to identify putative lanthipeptide precursors by fetching the small peptide open reading frame (orf) adjacent to *lanM* genes [27]. A total of 348 putative precursors associated with 103 LanM proteins (Supplementary Table 1 and Supplementary data file) were selected based on the presence of both Ser/Thr and Cys residues in their C-terminal core peptides, which are indispensable for forming the characteristic thioether crosslinks of lanthipeptides, and subjected to sequence similarity network (SSN) analysis (Fig. 1b, c). None of the archaeal class II lanthipeptide precursors clustered with these of bacterial origins in the SSN analysis, highlighting the potential for discovering unique chemistry from archaeal lanthipeptide BGCs (Fig. 1b). Notably, our further SSN analysis of archaeal lanthipeptide precursors not only revealed some identical and similar homologies of bioinformatically identified families Halolancins (HloA), Haladacins (HldA), Halobiforcins (HlbA), and Haloferaxcins (HlfA) in Castro et al. [5] (Fig. 1c) but also suggested new families (e.g., clusters 4–10) with unique sequence space of class II lanthipeptide. Most of these predicted precursors within the same cluster were conserved in both C- and N-terminal regions (Supplementary Fig. 5). It is worth mentioning that none of these reported BGCs and their corresponding lanthipeptides had been experimentally verified when we initiated the present study. Our preliminary analysis of biosynthetic potential suggested that archaeal SMs, particularly class II lanthipeptides, are diverse and untapped, providing a good starting point for further discovery and biosynthetic study of archaeal natural products.

### Linking the biosynthetic loci of class II lanthipeptides to metabolites via metabolic analysis

The diverse lanthipeptide BGCs were of particular interest, as they typically encode antimicrobials, which are envisioned to mediate social and competitive interactions within the bacterial community [28, 29]. We sought to chemically investigate archaeal lanthipeptides and study their roles in archaeal antagonistic interactions. We focused on halophilic archaea from Haloarchaea for archaeal lanthipeptides discovery based on three reasons: (i) Haloarchaea are well-known for their antagonistic interactions in the halophilic niche, while the antagonistic origin of these interactions remains unknown [5, 13–17]; (ii) cultivated Haloarchaea is an exceptionally well-suited model for the study of archaeal biology [30]; (iii) Haloarchaea harbor diverse uncharacterized lanthipeptide BGCs (Supplementary Figs. 1–5, and Supplementary data file). Guided by genomic analysis, five available isolates (*Haloferax mediterranei* ATCC33500, *Halorussus litoreus* HD8-51, *H. larsenii* JCM13917, *H. salinus* YJ-37-H, and *Halomicrobium mukohataei* DSM12286) that harbor lanthipeptide BGCs (Supplementary Fig. 6 and Supplementary data file) were selected, cultured, and subjected to mass spectrometry (MS)-based metabolic analysis.

Matrix-assisted laser desorption/ionization-time of flight (MALDI-TOF) mass spectrometry (MS) analysis of metabolic profile showed that three strains (*H. larsenii* JCM13917, *H. salinus* YJ-37-H, and *H. mukohataei* DSM12286) exhibited diverse typical peptide signals (molecule weight > 800) (Supplementary Figs. 7–9), hinting the secondary metabolic potential of Haloarchaea species. Further analysis of the metabolites produced by these strains was performed using high-resolution (HR) LC–MS and the MS/MS-based molecular networking [31] via the Global Natural Product Social (GNPS) platform [32]. Notably, *H. salinus* YJ-37-H that harbors six lanthipeptide BGCs (Supplementary Fig. 6) was found to be a prolific producer, particularly rich in diverse peptidic metabolites (Supplementary Figs. 8 and 10).

**Fig. 2:**
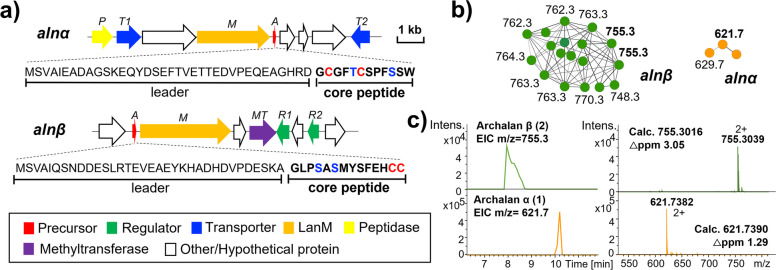
Metabolic analyses reveal new lanthipeptides from archaea. **a**The BGCs and precursor peptides of archalans α (**1**) and β (**2**) in *Halorussus salinus* YJ-37-H. **b** Peptide signals corresponding to those two lanthipeptide BGCs were picked out from the MS/MS networking of *H. salinus* YJ-37-H. Nodes are labeled with the monoisotopic precursor ion *m/z* values. **c** The HRMS spectra of archalans α and β from the two clusters in **b**

To link the metabolic profile of *H. salinus* YJ-37-H to its genetic context, we mapped the observed HRMS signals to calculated *m/z* values of bioinformatically predicted core peptides with anticipated modifications including dehydration, methylation, and dehydrogenation (e.g., disulfide crosslink), etc. We found two clusters of peptidic HRMS signals that matched well with the core peptides of two anticipated lanthipeptide BGCs: *alnα* and *alnβ* (Fig. 2a–c and Supplementary Tables 2 and 3). Additionally, the HR-MS/MS analysis of compound **1** (*m/z*: 621.7) revealed the same SW motif observed in the C-terminal core peptide (GCGFTCSPFSSW) encoded by *alnα*. Similarly, the HR-MS/MS analysis of compound **2** (*m/z*: 755.3) revealed the same linear methylated GLP motif found in the N-terminal of *alnβ*’s core peptide (GLPSASMYSFEHCC) (Supplementary Table 3). Altogether, we suggested that compounds **1**–**2** and their corresponding analogs (Fig. 2b-c and Supplementary Table 3) are RiPPs encoded by class II lanthipeptide BGCs *alnα* and *alnβ* (Fig. 2a and Supplementary Table 2), respectively, which were not previously predicted by any bioinformatic analysis. Guided by the bioinformatically identified precursor peptide sequences and accompanying MS data, we successfully linked these putative lanthipeptides to their biosynthetic loci.

### Discovery of two new lanthipeptides from archaea: archalans α-β

**Fig. 3:**
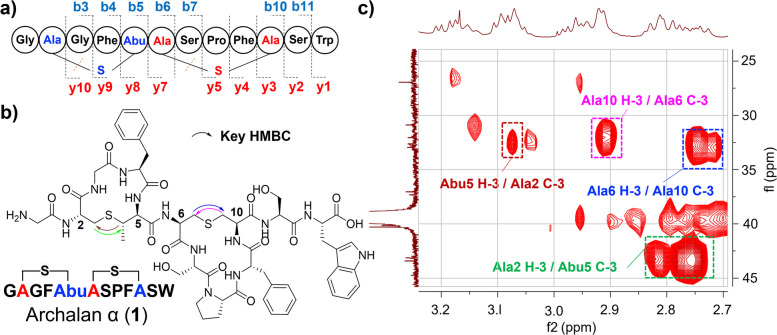
New lanthipeptide archalan α (**1**) isolated and identified from *Halorussus salinus* YJ-37-H*. ***a** The amino acid sequence of **1** with verified C-S bonds and marked b/y ions. **b** The chemical structure of **1** with key HMBC correlations for MeLan and Lan subunits showed in **c**

We next sought to elucidate the structures of compounds **1** and **2** by the MS/MS and NMR analysis. Mass signals at *m/z* 621.7382 [M + 2H]^2+^, indicative of the molecular formula C_57_H_71_N_13_O_15_S_2_ (△ + 1.29 ppm) of compound **1**, matched with the predicted core peptide of BGC *alnα* with two dehydration modifications, which was also further supported by MS/MS fragmentation analysis (Supplementary Fig. 11). The MS/MS fragmentation pattern matched well with the core peptide, except that Cys2 and Cys6 turned out to be Dha with a mass loss of 34 Da, while Thr5 and Ser10 residues were observed with a mass increase of 16 Da, due to the breaking of the C_γ_-S bond in Cys residues during fragmentation (Fig. 3a, Supplementary Table 3 and Supplementary Fig. 11). These results suggested the installation of the thioether-bridged amino acids, lanthionine (Lan) crosslinking Cys6 and Ser10 and methyllanthionine (MeLan) between Cys2 and Thr5. To fully characterize the structure of **1**, we purified 2.0 mg of **1** from a 20 L culture and elucidated its structure using extensive NMR analysis (Fig. 3b–c, Supplementary Figs. 12–14, 23 and Supplementary Table 4). The observation of many exchangeable amide NH protons (*δ*_H_ 7.8–10.8 ppm) occurred in the ^1^H-NMR and carbonyl carbons (*δ*_C_ 165–173 ppm) in ^13^C-NMR spectra supported the peptidic nature of **1**. Compound **1** was further deduced to contain 2 × Gly, 2 × Phe, 2 × Ser, 1 × Pro, 1 × Trp, 1 × Lan, and 1 × MeLan through HSQC, COSY, and HMBC data. HMBC correlations found within MeLan or Lan subunits further supported the installation of methyllanthionine and lanthionine motifs crosslinking Cys2 and Thr5, Cys6 and Ser10, respectively (Fig. 3c and Supplementary Table 4). The planar structure of **1** confirmed the dehydration of genetically encoded Thr5 and Ser10 and subsequent addition of Cys2 and Cys6 to the transients Dhb-5 and Dha-10, respectively. To determine the absolute configurations of all amino acid residues, we conducted advanced Marfey’s analysis [33] for **1** using L/D-FDLA (5-fluoro-2,4-dinitrophenyl-L/D-leucinylamide) (Supplementary Table 5). Results showed that all unmodified amino acids existed as L-configuration. For two thioether rings, the “D before L” was observed for the FDLA derivatives of MeLan and “L before D” was observed for the FDLA derivatives of Lan in **1**, which was consistent with the previously reported Marley’s analysis of MeLan (DL) and Lan (LL) [34, 35]. Taken together, we assigned a DL absolute configuration for MeLan and an LL for Lan. This newly identified class II lanthipeptide, named archalan α (**1**), is the first lanthipeptide identified from archaea to the best of our knowledge.

**Fig. 4:**
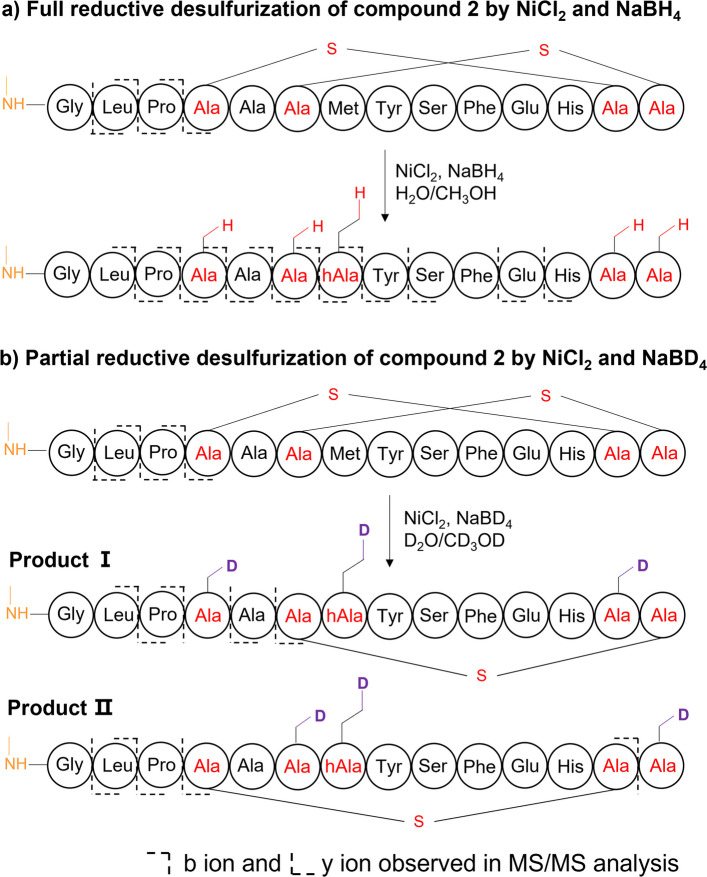
Characterization of crosslinks in archalan β (**2**). **a**–**b** Characterization of thioether crosslinks in **2** by full or partial reductive desulfurization, followed by MS/MS analysis of the resulting products

We next resorted to a combination of chemical derivatization (e.g., desulfurization by NiCl_2_ and NaBH_4_/NaBD_4_) and MS^n^ analysis to infer the planar structures of **2** encoded by BGC *alnβ* (Fig. 4 and Supplementary Figs. 15–19), due to their limited amount. Compound **2** gave a prominent doubly charged [M + 2H]^2+^ (*m/z*, 755.3012) peak by HRMS for C_66_H_92_N_16_O_19_S_3_ (△ + 0.53 ppm). Compared to the unmodified core peptide, the observed *m/z* of **2** had a mass loss of 22.0064 Da, matching well with two dehydrations (− 2H_2_O, − 36.0211 Da) and one methylation (+ CH_2_, + 14.0157 Da) at the N-terminal of Gly1 (Supplementary Table 3), which were further supported by the tandem MS analysis of **2** (Supplementary Fig. 16). Deduction of methylation at the N-terminal of Gly1 was also supported by the association of a methyltransferase gene found in the BGC *alnβ*. Two dehydrations due to the formation of C-S crosslinks were further supported by a tandem MS analysis of the full reduction-desulfurization product (Fig. 4a and Supplementary Fig. 17). To further identify its ring topology, **2** was partially reduced and desulfurized following an established protocol [34], generating two different partial desulfurized productions (Fig. 4b, Supplementary Figs. 18–19). According to MS/MS analysis, the first one showed an intact Ser6/Cys14 crosslink and a deuterated Ala reduced from Ser4, and the other showed an intact Ser4/Cys13 crosslink and a deuterated Ala reduced from Cys14. Together, **2** was identified as a class II lanthipeptide, named archalan β, likely containing one methylation at the N-terminal of Gly1 and two C-S crosslinks with intertwined topology (Fig. 4).

With the characterized chemical structures of archalans in hand, we attempted to bioinformatically analyze the chemical feature and diversity of archaeal lanthipeptides based on different characteristics, such as the diversity of precursor peptides, the number of putative dehydrations, and the number, size, and topology of the thioether rings. Firstly, the precursors and the core peptides are significantly shorter than their known bacterial counterparts (Supplementary Fig. 20). Secondly, their core regions were also highly diverse and distinct from their bacterial counterparts, exhibiting different topologies compared to other known lanthipeptides. In particular, the ring systems of lanthipeptides even from the same host were diverse, ranging from simple non-overlapping ‘bicycle’ rings exemplified by **1** to highly complex, intertwined topology deduced by **2** (Supplementary Figs. 21–22). In brief, archaeal lanthipeptides were short in peptide length but highly diverse in the amino acid residues of the core peptide. Additionally, the ability to form diverse lanthionine rings even within the same producing host further diversified archaeal lanthipeptide structural diversity, which endow archaea with multiple chemical options for niche adaptation.

### Archalan α exhibits anti-archaeal activity against closely related Haloarchaea

**Fig. 5:**
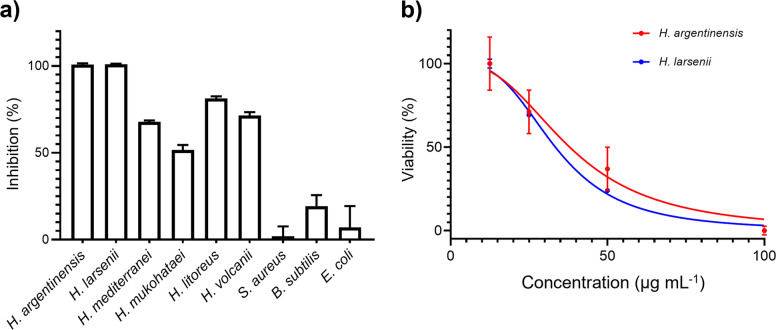
Anti-archaeal activity of archalan α against closely related Haloarchaea. **a** The antimicrobial activity of archalan α (100 μg mL^−^.^1^) against six haloarchaea and three bacteria strains. **b** Estimation of the half-maximal inhibitory concentration (IC_50_) of archalan α against *H. argentinensis* DSM 12282 and *H. larsenii* JCM13917. Data from three repeats (mean ± SEM) are shown. The IC_50_ value was determined by non-linear regression using GraphPad Prism 8

We next sought to investigate whether they contribute to archaeal antagonistic interactions. We tested purified archalan α (**1**) in two assays: anti-archaeal against six closely related Haloarchaea and antibacterial against three bacteria strains. No significant activity was observed in the antibacterial assay with inhibition less than 30% at 100 μg mL^−1^ (Fig. 5a). In contrast, archalan α showed significant inhibitory activity against several closely related Haloarchaea species. In particular, **1** is potent against extremely halophilic isolates of *H. argentinensis* and *H. larsenii*, with IC_50_ of 33–38 μg mL^−1^ (Fig. 5b). Archalan α is the first class of anti-archaeal lantibiotics identified to the best of our knowledge. Being the products of Haloarchaea isolated from halophilic environments predominated by archaea [36], archalan α exhibited narrow-spectrum anti-archaeal activity against closely related halophilic archaea (Supplementary Table 6). These results suggested that archalan may play a role in archaeal antagonistic interaction in the halophilic niche. Like bacteria, archaea are capable of producing diverse secondary metabolites that may mediate competitive or cooperative interactions with their biotic and abiotic environments. Castro et al. instead concluded that the archaeal lanthipeptide BGCs are unlikely to contribute to the biosynthesis of the main antagonistic compounds in *H. mediterranei* ATCC33500 [5]. However, we believe that the lack of the isolated lanthipeptides or its undetected yield from wide-type strains renders the results inconclusive. Though the ecological function of these compounds is not yet fully understood, our genomic-guided discovery of anti-archaeal lanthipeptide paves the way for the discovery of more antagonistic SMs and poses an intriguing question: how archaea employ SMs to shape their microbiome communities?

## Conclusion

Diverse and highly variable systems involved in small molecule-mediated interactions are ubiquitous in bacteria but much less studied in archaea. Previously, our understanding of archaeal interactions was limited to genomic analysis or antagonistic assay among a limited number of archaea isolates [5, 10–15, 17]. In this study, we conducted a genomic analysis to obtain insight into the biosynthetic potential of archaeal SMs, which provides a starting point for the genomic-guided discovery of archaeal SMs. Based on the genomic and metabolic results, we defined one lanthipeptide with antagonistic activity from halophilic archaea. We fully characterized the chemical structure of antagonist archalan α, representing the first lantibiotic from archaea. This search resulted in the identification of a narrow-spectrum anti-archaeal lanthipeptide that was implicated in archaeal antagonism among closely related halophilic archaea. However, the analysis presented here cannot be considered exhaustive, and the genomic strategies employed to predict archaeal BGCs remain to be refined by knowledge accumulation of archaeal SMs and their biosynthesis. Additionally, how archaea employ lanthipeptides to shape their microbiome communities in the environmental niche remains to be studied. Nevertheless, our discovery of anti-archaeal lanthipeptide is expected to stimulate experimental research to advance the understanding of poorly characterized archaeal chemical biology. In addition to enhancing our understanding of the biosynthetic potential and the chemical diversity of archaeal SMs, the discovery of anti-archaeal lanthipeptide opens up exciting opportunities for future research toward various new ecological roles for archaea.

## Methods

### BGCs analysis

Although no specific bioinformatic tool for the prediction of archaeal BGCs has been developed yet due to the extreme scarcity of chemical and genetic information on archaeal SMs, the antiSMASH bacterial version has been successfully used to predict archaeal BGCs from the ocean and glacier microbiome [22, 26–28]. Publicly available archaeal genomes were downloaded from NCBI RefSeq and GenBank databases (accessed in Aug. 2021) and analyzed by antiSMASH 6.0 with default parameters [25]. Genomes were deduplicated by Mash distance with a cutoff of 0.004 (≥ 99.6% genome similarity) using Mash tool v2.3 [37]. Representative genomes were selected based on the priority of higher assembly level (from higher to lower: complete genome, chromosome, scaffold, contig) and lower contig count. CheckM [38] was used to check the completeness and contamination of metagenome-assembled genomes (MAGs), and MAGs with contamination > 5% were further discarded. BGC class was labeled as the product annotation from antiSMASH. The precursor peptides of class II lanthipeptide were predicted from those ten orf genes adjacent to *lanM* with length shorter than 100 amino acids and Cys and Thr/Ser within 20 amino acids of the C-terminal.

The minimal cosine distance of an archaeal BGC to 1910 BGCs from MiBiG 2.0 was served as its distance to known BGCs. BGC features were extracted by BiG-SLiCE version 1.1.0 with default parameters [39]. Pairwise cosine distances between archaeal BGCs were computed using the SciPy [40] library in Python 3.8. The minimal cosine distance of an archaeal BGC to 1910 BGCs from MiBiG 2.0 was served as its distance to known BGCs. GCF accumulation curve was generated with the specaccum function in vegan package. Archaeal BGCs with a distance > 0.2 were considered novel.

### Phylogenetic analysis of LanMs and LanAs

In Mega 11 [41] (v11.0.8), LanM sequences from archaeal lanthipeptide BGCs and eleven known bacterial LanMs were aligned with the ClustalW method. The resulting alignment was subjected to phylogenetic tree construction using the default Maximum Likelihood method. Detailed statistical parameters used to construct the phylogenetic tree were as follows: analysis, phylogeny reconstruction; statistical method, maximum likelihood; test of phylogeny, none; substitution type, amino acid; model/method, Jones-Taylor-Thornton (JTT) model; gaps/missing data treatment, use all sites; ML heuristic method, nearest-neighbor-interchange (NNI); and branch swap filter, none; no. of threads, 4. The tree was visualized by iTOL v6 [42]. Precursor peptide sequences were aligned by Mega 11 (v11.0.8) with the ClustalW method. The resulting alignment generated sequence logos visualized using WebLogo (v3.7.4) [43].

### SSN analysis of precursor peptides (LanAs)

A database containing 348 archaeal LanAs found in our study predicted with core and leader region by antiSMASH and 2972 reported bacterial LanAs [24] was built to construct the SSN [44]. All sequences were listed in the supporting information. An *E*-value of 1.0 × 10^−5^ and Alignment Score Threshold of 5 were used to define the similarity between the query LanAs. A sub-SSN of archaeal LanAs was built based on the *E*-value of 1.0 × 10^−1^ and Alignment Score Threshold of 10. The resulting SSN was visualized using Cytoscape 4.1 [45].

### Archaeal precursor topology analysis

Blastp was applied to identify similar sequences of archalans in all putative archaeal precursors. Core peptides that shared a similar conserved region with Cys and Ser/Thr residues with each archalan were considered to share a similar ring topology. ClustalW was used to recognize and display the conserved amino acids of those archalan homolog precursors.

### General materials, reagents, and strains

Biochemicals and media components for bacterial cultures were purchased from standard commercial sources. Sinapic acid, NaBH_4_, NaBD_4_, and NiCl_2_ were purchased from Sigma-Aldrich (USA). *Nα*-(2,4-dinitro-5-fluorophenyl)-L/D-leucinylamide (L/D-FDLA) was from TCI Development Co., Ltd. (China). Dithiothreitol was from Macklin (China) and nisin was from Aladdin (China). All strains used in this study are listed in Supplementary Table 6.

### General experimental procedures

1D and 2D NMR spectra were recorded at 298 K on a Bruker Avance DRX 600 FT-NMR spectrometer (500 and 125 MHz for ^1^H and ^13^C NMR, respectively). Matrix-assisted laser desorption/ionization-time of flight (MALDI-TOF) mass spectra were recorded on a Bruker Ultraflex II in positive ion mode using 10 mg mL^−1^ sinapic acid dissolved in 50% methanol as the matrix. High resolution-LCMS analyses were performed on UltiMate 3000 UHPLC Systems with a Waters Acquity UPLC BEH C18 column (1.7 μm, 130 Å, 2.1 × 150 mm) coupled to Bruker impact™ II mass spectrometer unless otherwise stated. The column was maintained at 40 °C and run at a 0.2 mL min^−1^ flow rate, using 0.1% formic acid in H_2_O as solvent A and 0.1% formic acid in acetonitrile as solvent B. A gradient was employed for chromatographic separation starting at 5% B for 2 min, then 5 to 95% B for 15 min, washed with 95% B for 4 min, and finally held at 5% B for 1 min. All the samples were analyzed in positive polarity, using data-dependent acquisition mode. All data were analyzed with Bruker Compass DataAnalysis 4.3.

### Metabolic analysis by HR-LCMS

Five Haloarchaea strains were cultivated in 250-mL flasks containing 80 mL DSMZ medium 589 with 20 g L^−1^ sucrose for 5 days (37 °C and 200 r.p.m.). One milliliter culture broth of each strain was extracted from Diaion® HP-20 resin (Sigma-Aldrich), washed three times with 1 mL of H_2_O, and then collected crude extracts with elution of 1 mL MeOH. After being dried by a vacuum concentrator, all crude extracts were dissolved in methanol and subjected to MALDI-TOF and HR-LCMS.

### Lanthipeptide production, extraction, and isolation

*H. salinus* YJ-37-H was further cultivated in forty 2.5-L flasks containing 500 mL DSMZ medium 589 with 20 g L^−1^ sucrose for 5 days (37 °C and 200 r.p.m.). The culture supernatant was collected and extracted by HP20 resin, washed three times with 500 mL H_2_O, and finally eluted with 500 mL of MeOH three times. The elution was evaporated and subjected to an HPLC system (Waters, Parsippany, NJ, USA) for compound purification on Phenomenex Luna C18 column (250 mm × 10 mm, 5 μm, 100 Å) with a gradient method from 5 to 95% ACN/H_2_O containing constant 0.1% trifluoroacetic acid in 5–70 min at the flow rate of 3 mL min^-1^. Fourteen HPLC fractions were collected, evaporated, and then dissolved in methanol for LCMS. Compound **1** (~ 2 mg) was further purified from fraction 12 (retention time: 57–58 min). Compound **2** was detected in fraction 8 (39–43 min).

### GNPS networking

MSConvert [46] was used to convert all the MS datasets of crude extracts according to the instruction before uploading them to the Global Natural Product Social (GNPS) molecular networking project (http://gnps.ucsd.edu) [31]. For molecular networking, the minimum cosine score was set as 0.7. The parent ion mass tolerance was set to 0.02 Da and the fragment ion mass tolerance to 0.02 Da. Minimum matched fragment peaks were set to 6, minimum cluster size to 1 (MS Cluster off), and the library searches minimum matched fragment peaks to 6. When the analog search was performed, the cosine score threshold was 0.7 and the maximum analog search mass difference was 100. Molecular networks were visualized with Cytoscape version 3.8 [45] and manually excluded the signals falsely identified as archaeal metabolites.

### Structure elucidation

^1^H, ^13^C, ^1^H-^1^H-COSY, ^1^H-^13^C-HSQC, and ^1^H-^13^C-HMBC NMR spectra for compound **1** were recorded on Avance DRX 600 FT-NMR spectrometer (500 and 125 MHz for ^1^H and ^13^C NMR, respectively) using DMSO-*d*_6_. Chemical shifts were reported using the DMSO-*d*_6_ resonance as the internal standard for ^1^H-NMR DMSO-*d*_6_: *δ* = 2.50 p.p.m. and ^13^C-NMR DMSO-*d*_6_: *δ* = 39.6 p.p.m. Amino acid configurations of compound **1** were determined using the advanced Marfey’s method [33]. Briefly, compound **1** (~ 0.2 mg) was dissolved in 2 mL of 6 N HCl (with 5% (v/v)) heated at 110 °C for 14 h. The thioglycolic acid for avoiding the degradation of Trp was used. The hydrolysate was evaporated to dryness and dissolved in 100 μL of water, and aliquoted into two portions. Each portion was treated with 20 μL of NaHCO_3_ (1 M) and 50 μL of 1-fluoro-2,4-dinitrophenyl-5-L-leucinamide (L-FDLA) or D-FDLA (1 M) at 40 °C for 2 h, then quenched with 20 μL HCl (1 M) and dried under air. The mixtures were dissolved in 400 μL of MeOH for UPLC-MS analysis (Waters ACQUITY H-Class UPLC system coupled with an ACQUITY SQ detector 2 mass spectrometer). Separations were carried out on a Waters Acquity UPLC BEH C18 column (2.1 mm × 150 mm ID, 1.7 μm) by using a gradient elution mode at a flow rate of 0.2 mL min^-1^: 5–80% ACN/H_2_O with constant 0.1% trifluoroacetic acid in 2–20 min, 80–100% in 20–25 min, then isocratic 100% for 4 min. The stereochemistry was determined by comparing the retention time of L/D-FDLA derivatized amino acids. The hydrolysate of nisin was used as a reference to confirm the stereochemistry of the MeLan residue of compound **1**. For the configuration determination of Trp, HR-LCMS was applied using the same gradient with a UPLC CSH C18 column (1.7 μm, 130 Å, 2.1 × 100 mm). The standard L-Trp-L/D-FDLA were used as references to confirm the stereochemistry of Trp.

### Reductive desulfurization and LC–MS characterization of archalan β (**2**)

The HPLC fraction 8 containing archalan β (**2**) (F8, 39–43 min, ~ 3.0 mg) was suspended in 4.0 mL of CH_3_OH/H_2_O (1:1) or CD_3_OD/D_2_O (1:1), to which 20 mg of NiCl_2_ and 20 mg of NaBH_4_/NaBD_4_ were added. This mixture was stirred under 1 atm of H_2_ at room temperature for 2 h for partial desulfurization and 8 h for total desulfurization. Then, the mixture was centrifuged, and the supernatant was collected. A mixed solvent of CH_3_OH/H_2_O (ration = 1:1, 1.0 mL) was added to the black nickel boride pellets, and the suspension was sonicated and re-centrifuged to recover any residual peptide. Combined supernatants were dried under vacuum and stored at – 20 °C before HR-LCMS and MS^n^ analyses. The partial reduction supernatants were analyzed on LTQ Orbitrap Velos mass spectrometer (Thermo Scientific, San Jose, CA, USA).

### Anti-microbial activity of archalan α

Five indicator Haloarchaea strains, including *H. argentinensis*, *H. larsenii*, *H. mediterranei*, *H. mukohataei*, and *H. litoreus* HD8-51, were cultured in modified DSMZ medium 589 (with 20 g L^−1^ sucrose), while *H. volcanii* was cultivated in the 18% modified growth medium (144 g L^−1^ NaCl, 18 g L^−1^ MgCl_2_·6H_2_O, 21 g L^−1^ MgSO_4_·7H_2_O, 4.2 g L^−1^ KCl, 5 g L^−1^ peptone, 1 g L^−1^ yeast extract) [47]. Three indicator bacterial strains, *Bacillus subtilis* 168, *Staphylococcus aureus* ATCC25923, and *Escherichia coli* DH5α, were incubated in LB medium at 37 °C. Archaea were grown 24 h to stationary phase and adjusted in modified DSMZ medium 589 (with 20 g L^−1^ sucrose) to 5.0 × 10^5^ c.f.u. mL^−1^ in the wells of 96-well microtiter plates (Corning, USA), mixed with varying concentrations of compound 1 and incubated in the shaking incubator (37 °C and 200 r.p.m.) for 48 h. Bacteria (*B. subtilis* 168, *S. aureus* ATCC25923, and *E. coli* DH5α) were grown overnight (37 °C and 200 r.p.m.) to stationary phase and adjusted in LB broth to 5.0 × 10^5^ c.f.u. mL^−1^ in the wells of 96-well microtiter plates, mixed with varying concentrations of **1** and incubated in the shaking incubator (37 °C and 200 r.p.m.) for 24 h. The volume was 100 μL in each well. The sample was prepared in a stock solution of 100 μg μL^−1^ in DMSO and serially diluted across a 96-well plate to a final concentration of 100, 50, 25, and 12.5 μg mL^−1^. The cell growth was monitored at OD_600_ (Varioskan Flash, Thermo Scientific, USA), and the minimal inhibitory concentration (MIC) of compound 1 against each strain was defined as the lowest compound concentration at which no observed archaeal or bacterial growth. The viability was calculated by the value of OD_600_ difference detected in the experimental group at 48 h and 0 h divided by that of the control group. The inhibition value was obtained by subscribing the viability value from 100%. The IC_50_ value was determined by non-linear regression using GraphPad Prism 8 following the manual instructions.

## Supplementary Information

**Additional file 1: Supplementary Figure 1.** The biosynthetic capacity of archaeal secondary metabolites. **Supplementary Figure 2.** BiG-SCAPE/CORASON analysis of 50 representative lanthipeptide BGCs from 1681 RiPP BGCs detected by antiSMASH. **Supplementary Figure 3.** Phylogenetic analysis of 50 representative LanMs found in archaea and their corresponding biosynthetic gene clusters. **Supplementary Figure 4.** Phylogenetic analysis of Maximum likelihood (ML) tree of LanMs. **Supplementary Figure 5.** Precursor logo sequences of clusters 1-12 in SSN analysis results. **Supplementary Figure 6.** Lanthipeptide BGCs of selected haloarchaea prioritized upon genomic analysis. **Supplementary Figure 7.** MALDI-TOF mass spectra of *Haloferax larsenii* JCM13917. **Supplementary Figure 8.** MALDI-TOF mass spectra of *Halorussus salinus* YJ-37-H. **Supplementary Figure 9.** MALDI-TOF mass spectra of *Halomicrobium mukohataei* DSM12286. **Supplementary Figure 10.** Metabolic analysis of selected haloarchaea strain *H. salinus* YJ-37-H prioritized upon genomic analysis. **Supplementary Figure 11.** LC-MS/MS spectrum of archalan α (**1**). **Supplementary Figure 12.** Chemical structure of archalan α (**1**) with key ^1^H-^1^H COSY and ^1^H-^13^C HMBC correlations. **Supplementary Figure 13.** Key COSY (a), HSQC (b), and HMBC (c) correlations within methyllanthionine subunit of archalan α (**1**) in *d*_6_-DMSO. **Supplementary Figure 14.** Key COSY (a), HSQC (b), and HMBC (c) correlations within lanthionine subunit of compound **1** in *d*_6_-DMSO. **Supplementary Figure 15.** Characterization of the C-S crosslinks in archalan β (**2**). **Supplementary Figure 16.** LC-MS/MS spectrum of archalan β (**2**). **Supplementary Figure 17.** LC-MS/MS spectrum of fully desulfurized archalan β (**2**). **Supplementary Figure 18.** LC-MS/MS spectrum of partial desulfurized archalan β (**2**) product I. **Supplementary Figure 19.** LC-MS/MS spectrum of partial desulfurized archalan β (**2**) product II. **Supplementary Figure 20.** Structure feature of class II lanthipeptide. **Supplementary Figure 21.** Topology of bacterial class II lanthipeptide. **Supplementary Figure 22.** Topology of archaeal class II lanthipeptide. **Supplementary Figure 23.** NMR spectra of archalan α (**1**) in *d*_6_-DMSO. **Supplementary Table 1.** List of putative precursors identified in this study and previous studies. **Supplementary Table 2.** Predicted genes within the biosynthetic gene clusters of *alnα* and *alnβ*. **Supplementary Table 3.** Observed and calculated mass values of putative archaeal lanthipeptides. **Supplementary Table 4.** NMR data for archalan α (**1**) in *d*_6_-DMSO (500 MHz ^1^H NMR, 125 MHz ^13^C NMR). **Supplementary Table 5.** Advanced Marfey’s analysis of archalan α (Retention times (in min) constituent amino acids derivatized with D/L-FDLA). **Supplementary Table 6.** Strains used in this study.

## Data Availability

All genomes used in this research were obtained from the NCBI Assembly database (https://www.ncbi.nlm.nih.gov/assembly), with a full list of accession numbers provided in Supplementary data file. All BGCs and precursor sequences and their clustering information are in Supplementary File 1. LC–MS/MS data of all fractions from strain *H. salinus* YJ-37-H were deposited in the MassIVE Public GNPS data set (MSV000088831).
