# Supplementary material for: Genomic and metabolic analyses reveal antagonistic lanthipeptides in archaea
Source: Microbiome. 2023 Apr 14;11:74. doi: 10.1186/s40168-023-01521-1 (PMC10105419; doi:10.1186/s40168-023-01521-1)
Supplement: Supplementary file 2 — Additional file 1: Supplementary Figure 1. The biosynthetic capacity of archaeal secondary metabolites. Supplementary Figure 2. BiG-SCAPE/CORASON analysis of 50 representative lanthipeptide BGCs from 1681 RiPP BGCs detected by antiSMASH. Supplementary Figure 3. Phylogenetic analysis of 50 representative LanMs found in archaea and their corresponding biosynthetic gene clusters. Supplementary Figure 4. Phylogenetic analysis of Maximum likelihood (ML) tree of LanMs. Supplementary Figure 5. Precursor logo sequences of clusters 1-12 in SSN analysis results. Supplementary Figure 6. Lanthipeptide BGCs of selected haloarchaea prioritized upon genomic analysis. Supplementary Figure 7. MALDI-TOF mass spectra of Haloferax larsenii JCM13917. Supplementary Figure 8. MALDI-TOF mass spectra of Halorussus salinus YJ-37-H. Supplementary Figure 9. MALDI-TOF mass spectra of Halomicrobium mukohataei DSM12286. Supplementary Figure 10. Metabolic analysis of selected haloarchaea strain H. salinus YJ-37-H prioritized upon genomic analysis. Supplementary Figure 11. LC-MS/MS spectrum of archalan α (1). Supplementary Figure 12. Chemical structure of archalan α (1) with key 1H-1H COSY and 1H-13C HMBC correlations. Supplementary Figure 13. Key COSY (a), HSQC (b), and HMBC (c) correlations within methyllanthionine subunit of archalan α (1) in d6-DMSO. Supplementary Figure 14. Key COSY (a), HSQC (b), and HMBC (c) correlations within lanthionine subunit of compound 1 in d6-DMSO. Supplementary Figure 15. Characterization of the C-S crosslinks in archalan β (2). Supplementary Figure 16. LC-MS/MS spectrum of archalan β (2). Supplementary Figure 17. LC-MS/MS spectrum of fully desulfurized archalan β (2). Supplementary Figure 18. LC-MS/MS spectrum of partial desulfurized archalan β (2) product I. Supplementary Figure 19. LC-MS/MS spectrum of partial desulfurized archalan β (2) product II. Supplementary Figure 20. Structure feature of class II lanthipeptide. Supplementary Figure 21. Topology of bacteria [file 40168_2023_1521_MOESM1_ESM.pdf]

Genomic and metabolic analyses reveal antagonistic lanthipeptide metabolites in archaea

Haoyu Liang[1,7], Zhiman Song[1,2,7], Zheng Zhong[1], Dengwei Zhang[1,3], Wei Yang[4], Le Zhou[1,3], Ethan A. Older[5], Jie Li[5], Huan Wang[6], Zhirui Zeng[3,4*] and Yong-Xin Li[1,3*]

[1] Department of Chemistry and The Swire Institute of Marine Science, The University of Hong Kong, Pokfulam Road, Hong Kong, China

[2] Chemistry and Chemical Engineering Guangdong Laboratory, Shantou 515031, China

[3] Southern Marine Science and Engineering Guangdong Laboratory (Guangzhou), Guangzhou, China

[4] Department of Ocean Science and Engineering, Southern University of Science and Technology, Shenzhen 518055, China

[5] Department of Chemistry and Biochemistry, University of South Carolina, Columbia, South Carolina, USA

[6] State Key Laboratory of Coordination Chemistry, Chemistry and Biomedicine Innovation Center of Nanjing University, School of Chemistry and Chemical Engineering, Nanjing University, Nanjing, China

[7] These authors contributed equally to this work.

[*]To whom correspondence may be addressed.

Email: zengzr@sustech.edu.cn; yxpli@hku.hk

Table of Contents

Supplementary Figure *4*

Supplementary Figure 1. The biosynthetic capacity of archaeal secondary metabolites 4

Supplementary Figure 2: BiG-SCAPE/CORASON analysis of 50 representative lanthipeptide BGCs from 1681 RiPP BGCs detected by antiSMASH 5

Supplementary Figure 3: Phylogenetic analysis of 50 representative LanMs found in archaea and their corresponding biosynthetic gene clusters 6

Supplementary Figure 4: Phylogenetic analysis of Maximum likelihood (ML) tree of LanMs 8

Supplementary Figure 5: Precursor logo sequences of clusters 1-12 in SSN analysis results 9

Supplementary Figure 6: Lanthipeptide BGCs of selected haloarchaea prioritized upon genomic analysis 10

Supplementary Figure 7: MALDI-TOF mass spectra of *Haloferax larsenii* JCM13917 11

Supplementary Figure 8: MALDI-TOF mass spectra of *Halorussus salinus* YJ-37-H 12

Supplementary Figure 9: MALDI-TOF mass spectra of *Halomicrobium mukohataei* DSM12286 13

Supplementary Figure 10: Metabolic analysis of selected haloarchaea strain *H. salinus* YJ-37-H prioritized upon genomic analysis 14

Supplementary Figure 11: LC-MS/MS spectrum of archalan α (1) 15

Supplementary Figure 12: Chemical structure of archalan α (1) with key ^{1}H-^{1}H COSY and ^{1}H-^{13}C HMBC correlations 16

Supplementary Figure 13: Key COSY (a), HSQC (b), and HMBC (c) correlations within methyllanthionine subunit of archalan α (1) in d_6-DMSO 17

Supplementary Figure 14: Key COSY (a), HSQC (b), and HMBC (c) correlations within lanthionine subunit of compound 1 in d_6-DMSO 19

Supplementary Figure 15: Characterization of the C-S crosslinks in archalan β (2) ..21

Supplementary Figure 16: LC-MS/MS spectrum of archalan β (2) 22

Supplementary Figure 17: LC-MS/MS spectrum of fully desulfurized archalan β (2)23

Supplementary Figure 18: LC-MS/MS spectrum of partial desulfurized archalan β (2) product I 24

Supplementary Figure 19: LC-MS/MS spectrum of partial desulfurized archalan β (2) product II 25

Supplementary Figure 20: Structure feature of class II lanthipeptide 26

Supplementary Figure 21: Topology of bacterial class II lanthipeptide 27

Supplementary Figure 22: Topology of archaeal class II lanthipeptide 28

Supplementary Figure 23: NMR spectra of archalan α (1) in d_6-DMSO 29

Supplementary Tables *32*

Supplementary Table 1. List of putative precursors identified in this study and previous studies 32

Supplementary Table 2. Predicted genes within the biosynthetic gene clusters of *alna*

and *alnβ*..41
Supplementary Table 3. Observed and calculated mass values of putative archaeal lanthipeptides...42
Supplementary Table 4. NMR data for archalan α (1) in d_6-DMSO (500 MHz ^{1}H NMR, 125 MHz ^{13}C NMR)...43
Supplementary Table 5. Advanced Marfey's analysis of archalan α (Retention times (in min) constituent amino acids derivatized with D/L-FDLA)..45
Supplementary Table 6. Strains used in this study...46

Supplementary Figure

Supplementary Figure 1. The biosynthetic capacity of archaeal secondary metabolites

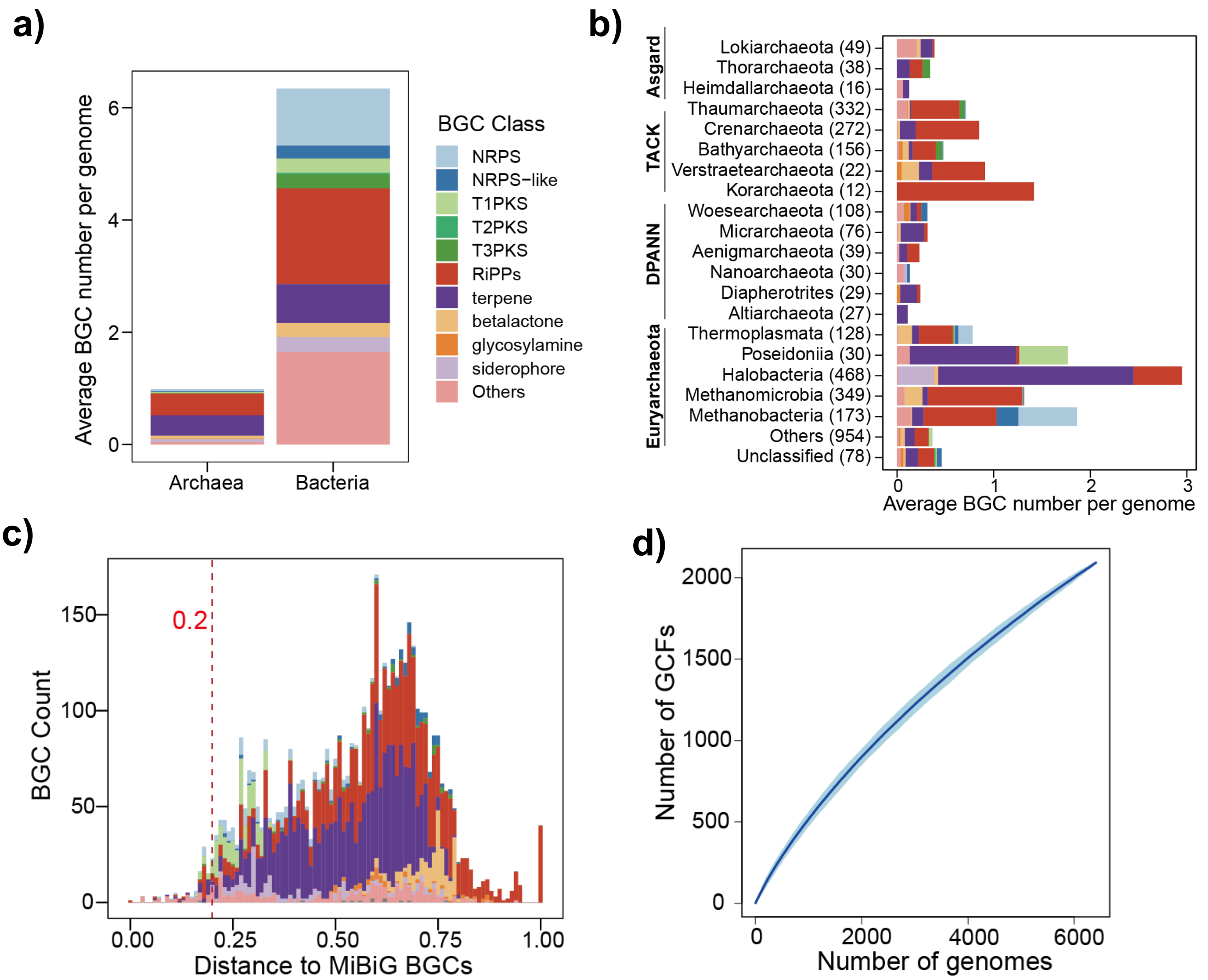

a) The estimated average number of antiSMASH-predicted BGCs per genome in archaea and bacteria. b) Distribution of BGCs across the archaea domain. Only non-MAG and MAGs with completeness > 80% of archaea were taken into account in (a) and (b). c) Distribution of cosine distance of 4803 BGCs to known BGCs from MIBiG. BGCs with cosine distance > 0.2 are considered new BGCs, accounting for 97.6% (4688 out of 4803). d) BGC family (GCF) accumulation curves. Further genome sequencing efforts of archaea would continually uncover additional novel BGC families. The blue shade area denotes the 95% confidence interval. Of note, there are unaddressed technical limitations in the preliminary BGC analysis of archaeal SM via the bacterial version antiSMASH. While we believe that the antiSMASH-based biosynthetic analysis provides valuable preliminary insight into the biosynthetic potential of archaea, the results represented here should be interpreted with caution.

Supplementary Figure 2: BiG-SCAPE/CORASON analysis of 50 representative lanthipeptide BGCs from 1681 RiPP BGCs detected by antiSMASH

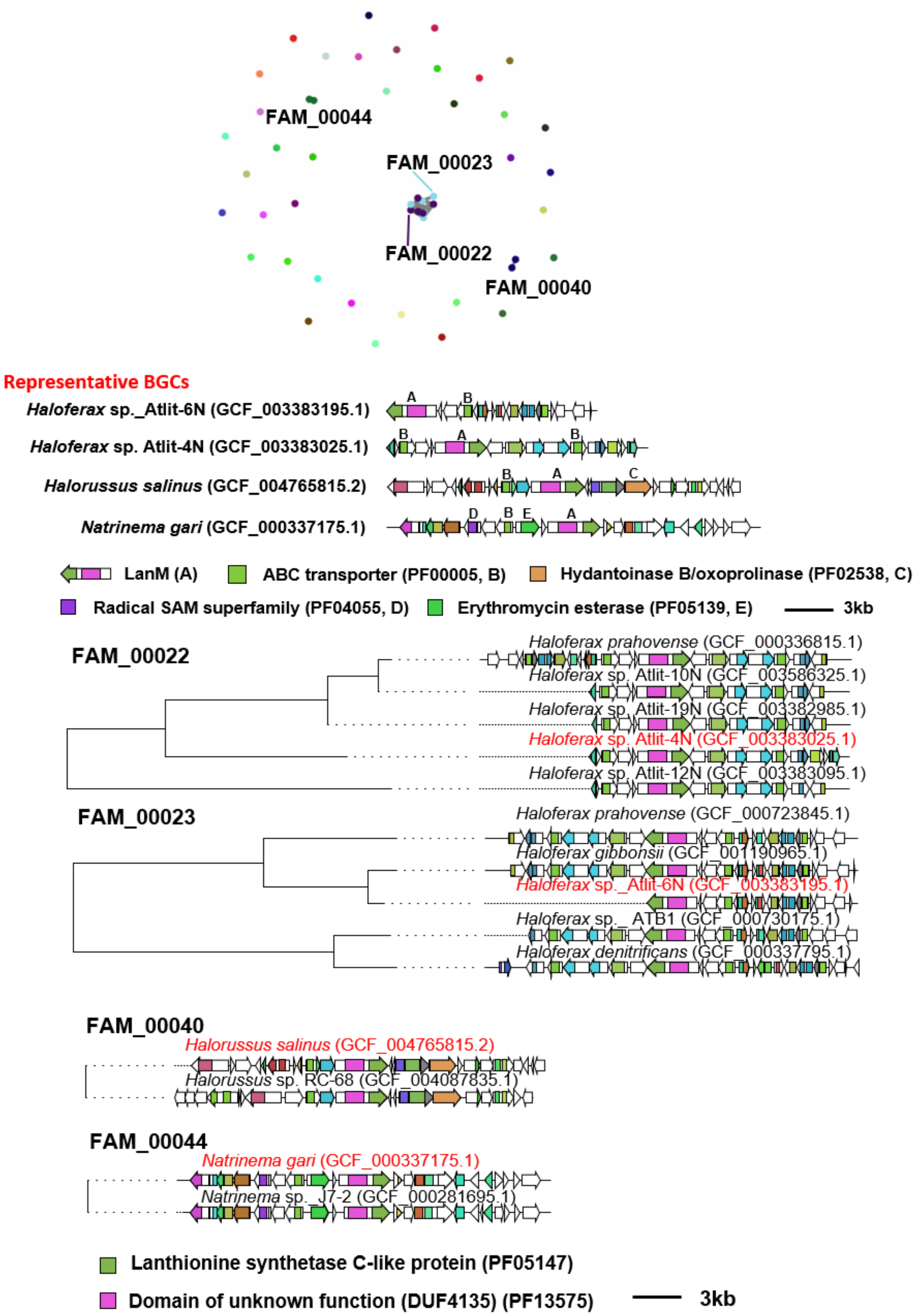

Among those 50 representative lanthipeptide BGCs, 48 BGCs were annotated to be class II lanthipeptides and only two class V lanthipeptides. All the representative BGCs were class II lanthipeptides containing gene *lanM* (Lanthionine synthetase C-like protein (PF05147) and domain of PF13575 and ABC transporter (PF00005).

Supplementary Figure 3: Phylogenetic analysis of 50 representative LanMs found in archaea and their corresponding biosynthetic gene clusters

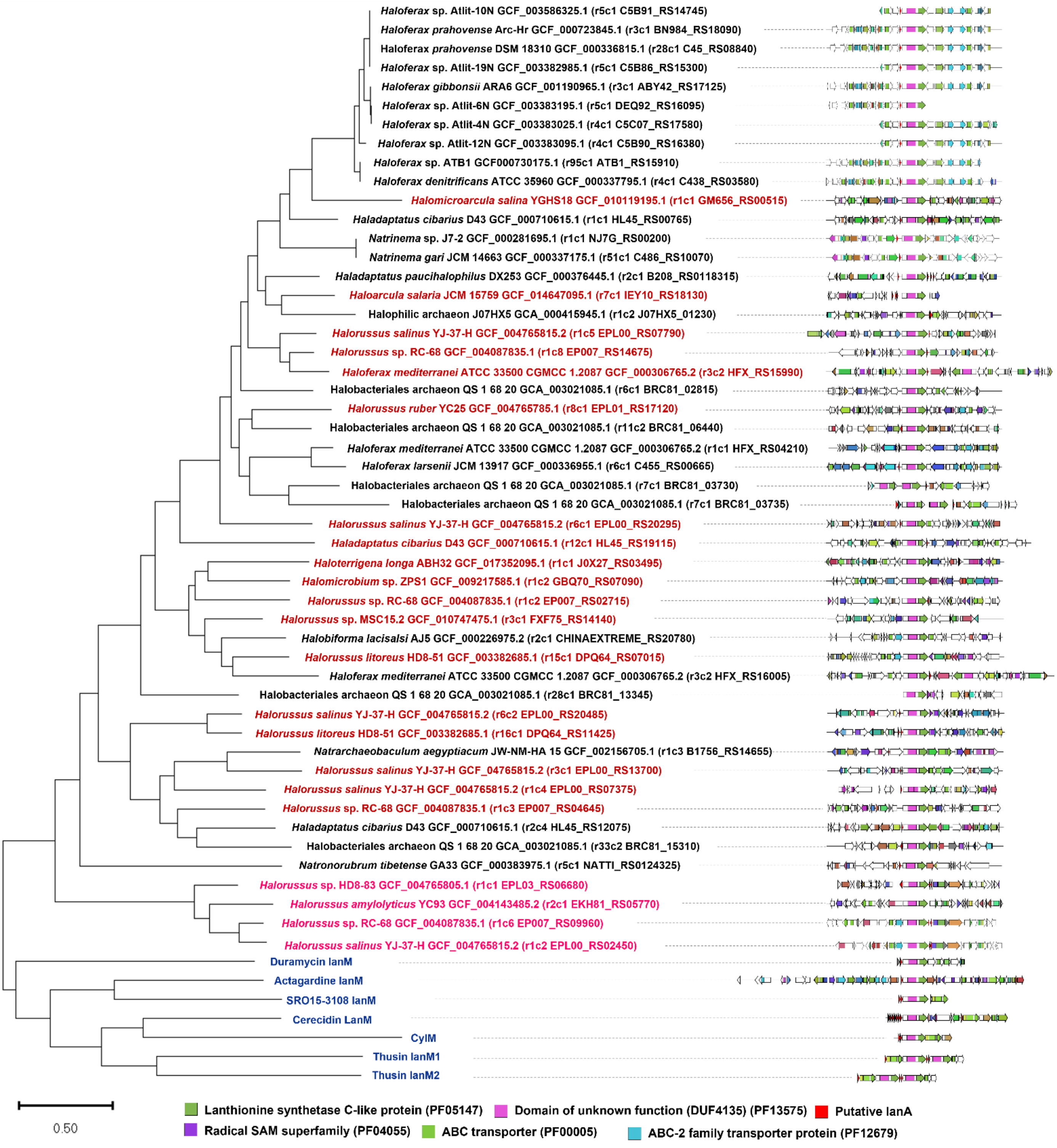

Left is the phylogenetic tree of 50 representative LanMs (identified from the genome with high completeness) in archaeal lanthipeptide BGCs with several bacterial LanM references (blue). Strain names in black represent those known LanMs found in both the previous study and our study, and red/pink represent new LanMs identified in this study. On the right is the respective lanthipeptide BGCs. The clade in pink is far away from other haloarchaea LanMs, representing novel class II lanthipeptide BGCs identified by antiSMASH. The 50 archaeal LanMs and 6 bacterial LanMs are in two different clades, indicating their sequence dissimilarity. Most BGCs have typical LanAs (red domain) and transporters (light green and light blue domains). Castro *et*

al. used NisB (ADJ56353) and CylM (AAK67266) as inquiry sequences to identify LanB and LanM-like enzymes with Position-Specific Iterated BLAST (PSI-BLAST)[1], which heavily relied on sequence similarity to known lanM enzymes. Limited by the phylogenetic closeness with inquiry sequence at some level, only 42 LanMs of 40 BGCs were found in the previous study, not including our experimentally verified BGCs in *Halorussus salinus* YJ-37-H. We applied antiSMASH-based analysis to obtain a total of 103 lanM containing BGCs (covering most of LanMs reported in Castro *et al*. 2021 and Walker *et al*. 2020), highlighting the feasibility of the antiSMASH approach in lanthipeptide BGC prediction.

Supplementary Figure 4: Phylogenetic analysis of Maximum likelihood (ML) tree of LanMs

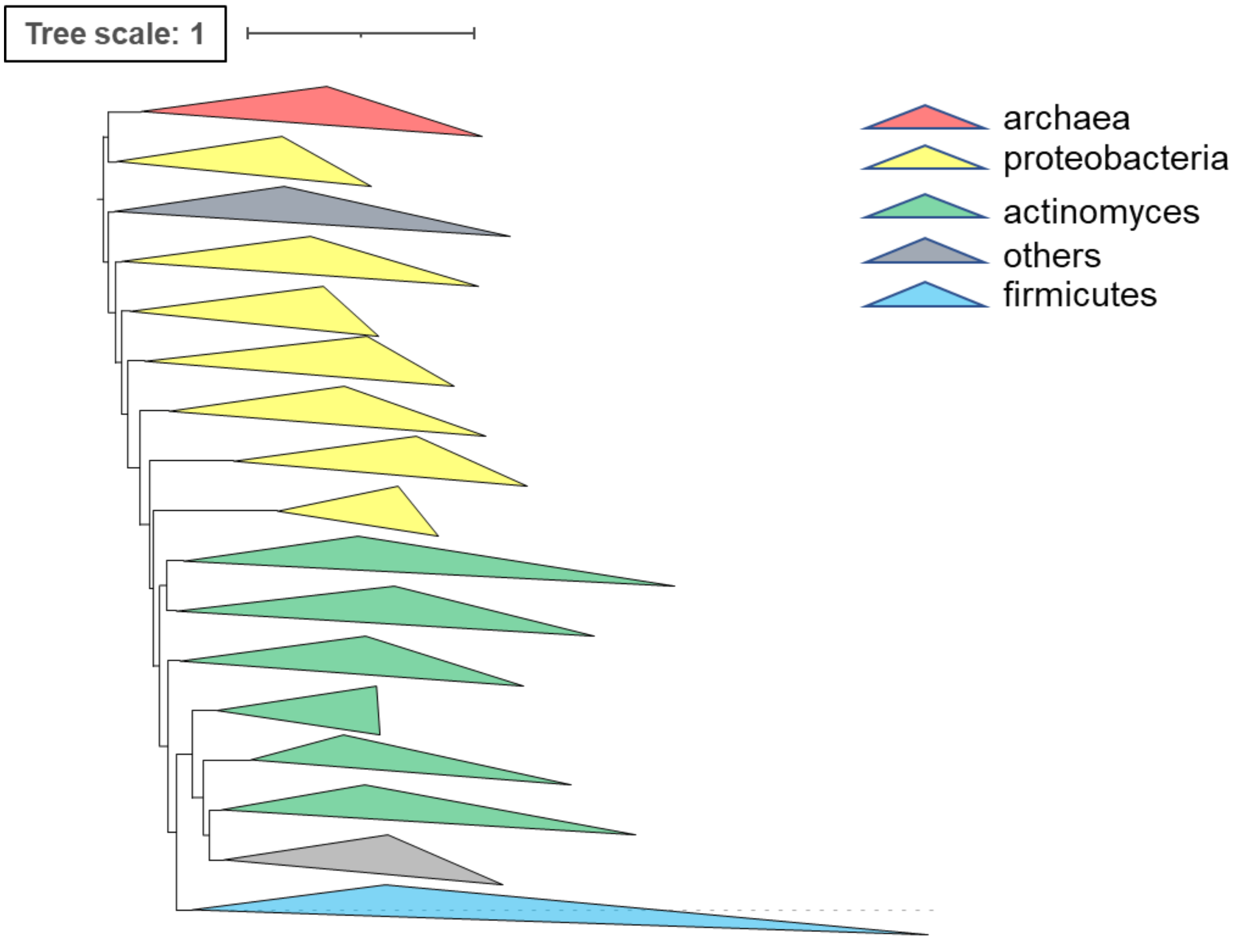

The ML tree exhibited the phylogenetic relationship of LanMs found in the phylum Euryarchaeota (red) and other bacterial LanMs. Archaeal LanMs were closely related to LanMs in proteobacteria.

Supplementary Figure 5: Precursor logo sequences of clusters 1-12 in SSN analysis results

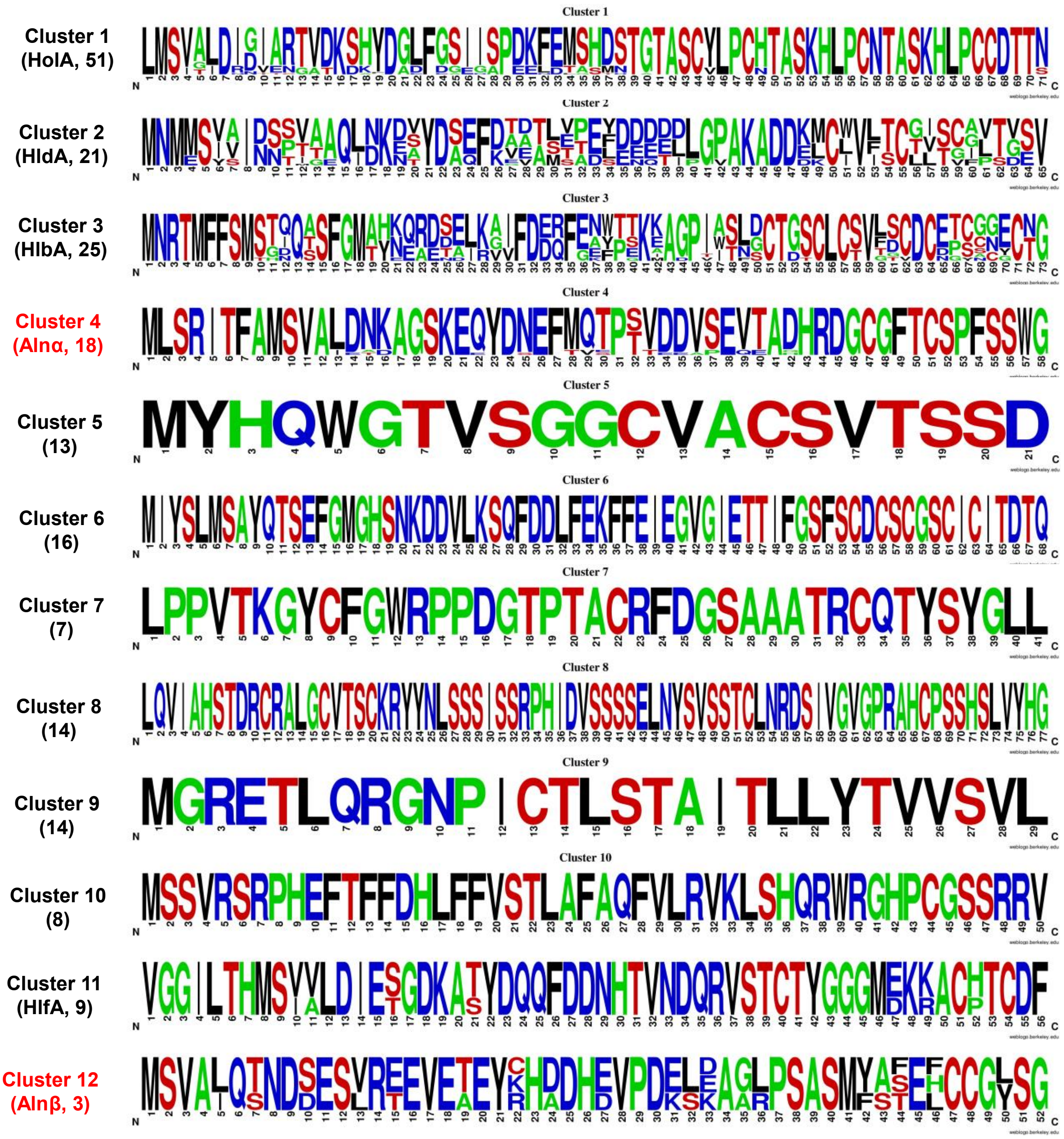

Clusters 1-3 and 9 were predicted to be homologies of Halolancins (HloA), Haladacins (HldA), Haloferaxcins (HlfA), and Halobiforcins (HlbA) as identified by Castro *et al.* in 2021[1], using ORFfinder and Rapid ORF Description & Evaluation Online (RODEO)[2]. Clusters 4 and 12 in red were experimentally identified in this study. The number in the figure represents for the number of sequences in the cluster and used for analysis. Except for clusters 2 and 3, precursors of other clusters were conserved in both C terminal and the N terminal regions. Of note, clusters 5 and 9 were shorter than other clusters with typical Ser/Thr and Cys residues.

Supplementary Figure 6: Lanthipeptide BGCs of selected haloarchaea prioritized upon genomic analysis

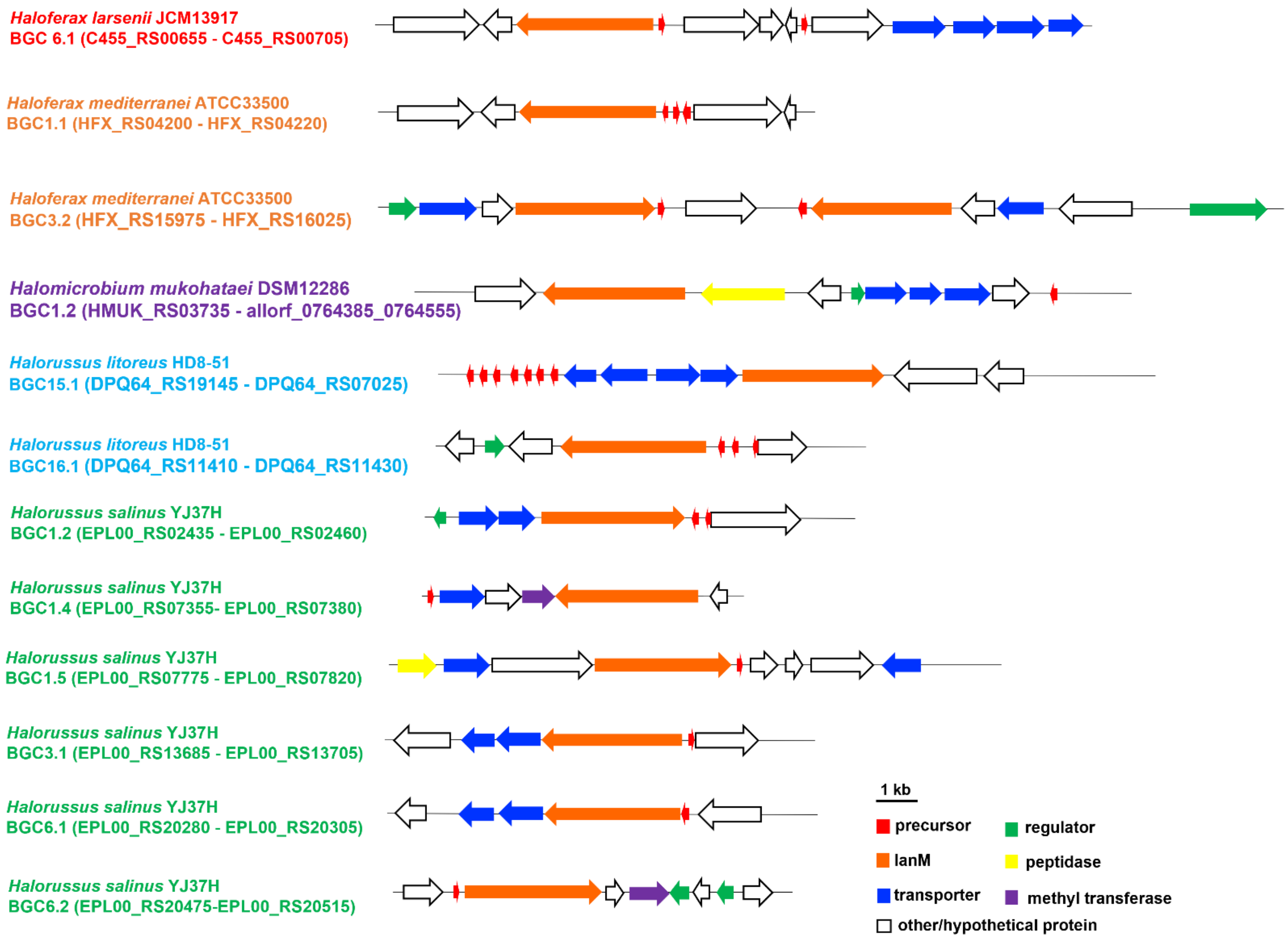

A total of twelve lanthipeptide BGCs annotated in five selected haloarchaea strains. Different colors represent different strains. *Halorussus salinus* YJ-37-H harbored six lanthipeptide BGCs.

Supplementary Figure 7: MALDI-TOF mass spectra of *Haloferax larsenii* JCM13917

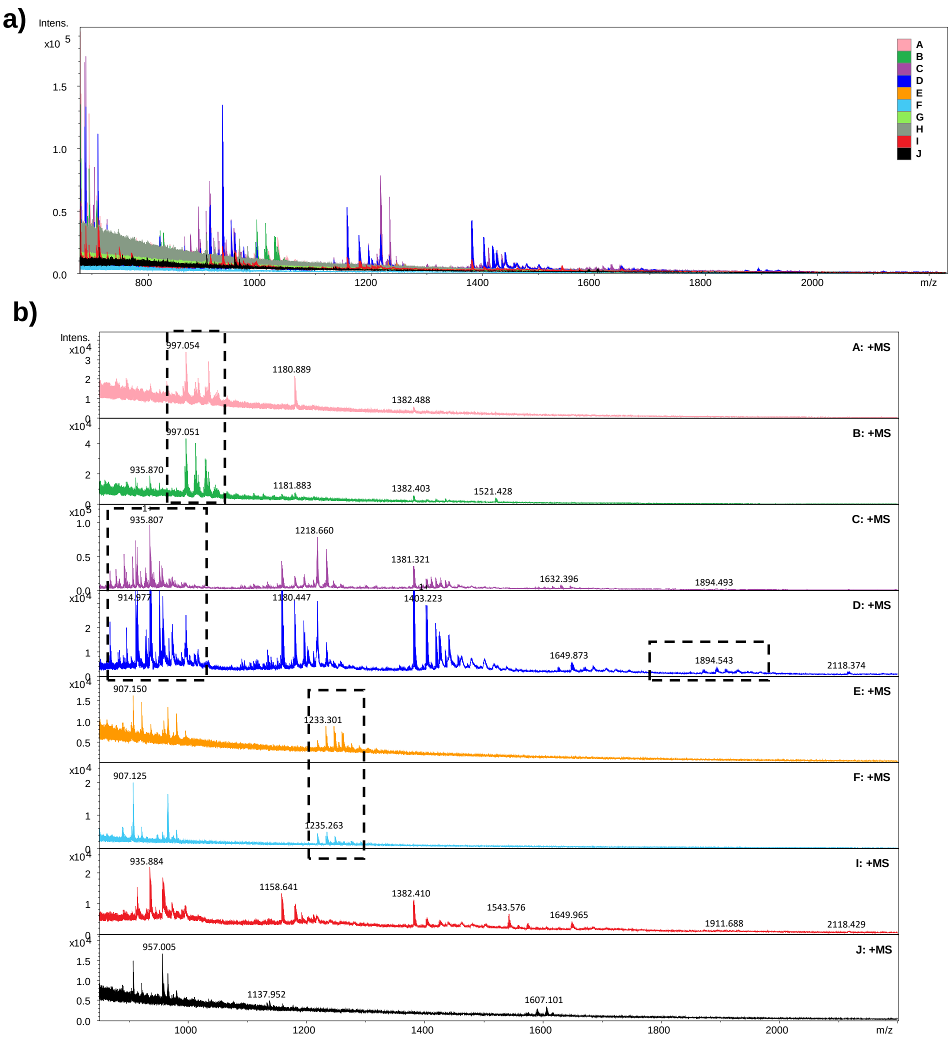

c)

Series	Temperature (°C)	Day (d)	Extraction method
A	37	3	Pellet, acetone, methanol
B	37	5	
C	30	3	
D	30	5	
E	37	3	Supernatant, HP-20, methanol
F	37	5	
G	30	3	
H	30	5	
I	Medium		Acetone, methanol
J	Medium		HP-20, methanol

Overlay of ten MALDI-TOF mass spectra (**a**) showing different fermentation conditions and extraction methods for strain *H. larsenii* JCM13917. Possible peptide signals (MV> 800) from the spectra above were listed and highlighted in black boxes **(b)**. Different culture conditions (**c**) for these strains include different temperatures (37 °C for A, B, E, F, and 30 °C for C, D, G, H) and different cultivation times (3 days for A, C, E, G, and 5 days for B, D, F, H). A-D are extracts of cell pellet extracted by acetone. E-H are extracts of supernatant extracted by HP-20 resin. I and J are negative control groups that are the crude extracts of medium extracted by acetone and HP-20 resin, respectively. Figure **c** also applies to Supplementary Figures 28 and 29.

Supplementary Figure 8: MALDI-TOF mass spectra of *Halorussus salinus* YJ-37-H

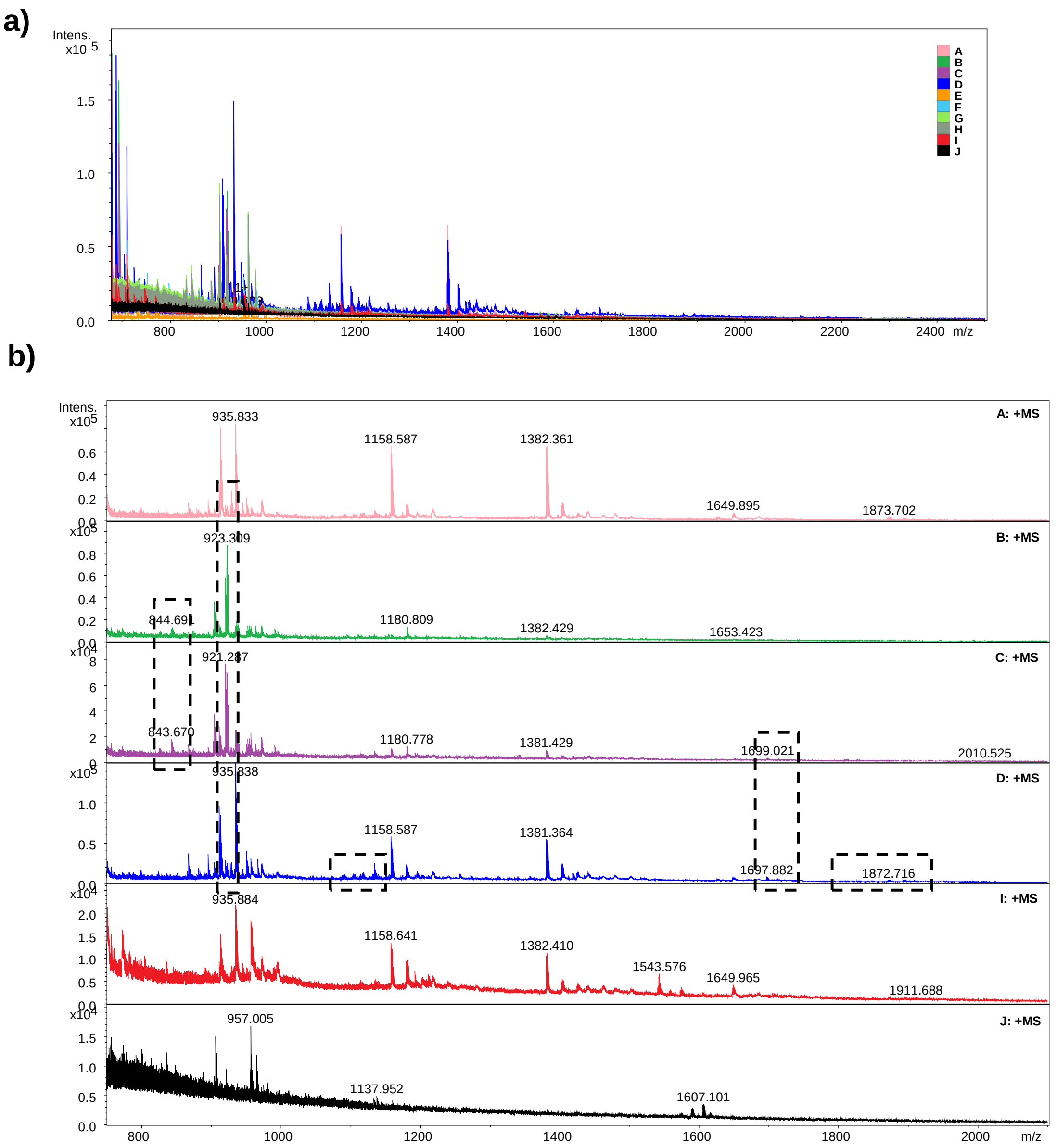

Overlay of ten MALDI-TOF mass spectra (**a**) showing different fermentation conditions and extraction methods for strain *H. salinus* YJ-37-H. Possible peptide signals (MV> 800) from the spectra above were listed and highlighted in black boxes **(b)**.

Supplementary Figure 9: MALDI-TOF mass spectra of *Halomicrobium mukohataei* DSM12286

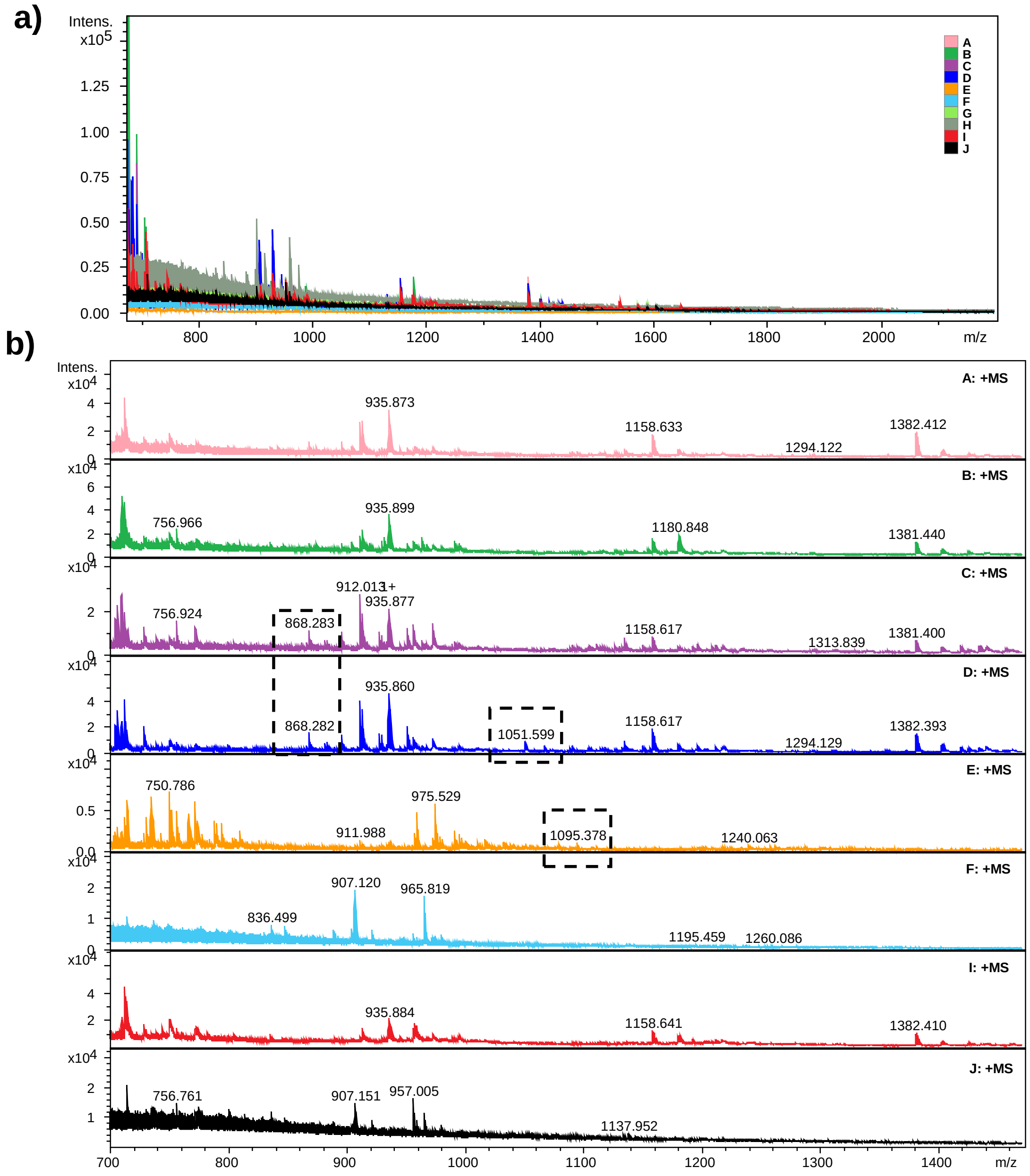

Overlay of ten MALDI-TOF mass spectra (**a**) showing different fermentation conditions and extraction methods for strain *H. mukohataei* DSM12286. Possible peptide signals (MV> 800) from the spectra above were listed and highlighted in black boxes **(b)**.

Supplementary Figure 10: Metabolic analysis of selected haloarchaea strain *H. salinus* YJ-37-H prioritized upon genomic analysis

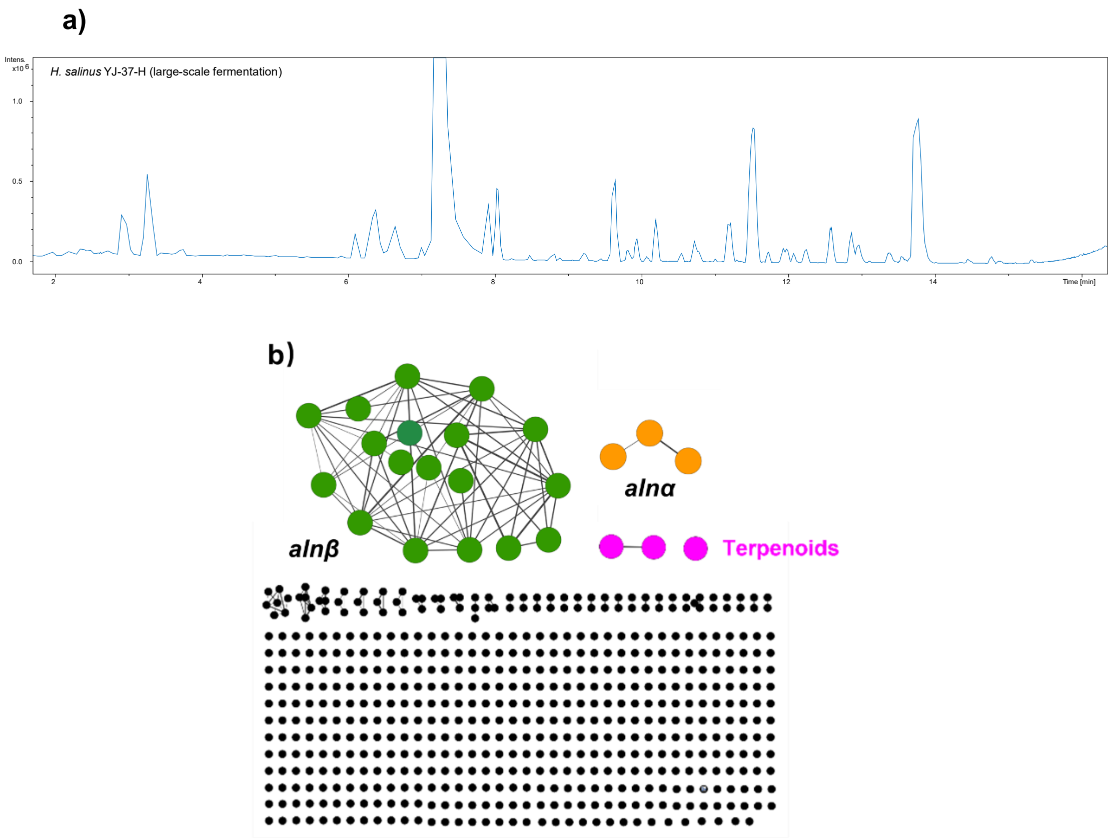

a) LC-MS profile of large-scale fermentation of *H. salinus* YJ-37-H. **b**) The molecular networking of *H. salinus* YJ-37-H generated by The Global Natural Product Social Molecular Networking (GNPS). The results exhibited the predicted products of genes *alnα*, *alnβ*, and the predicted terpenoids.

Supplementary Figure 11: LC-MS/MS spectrum of archalan α (1)

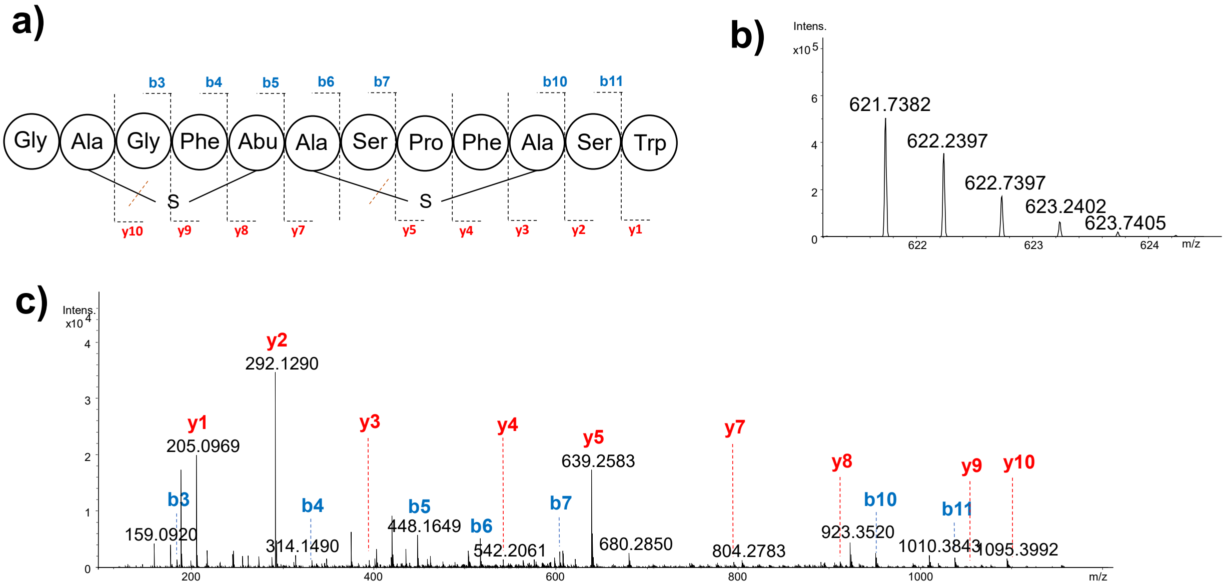

d)

Ion	Obs m/z	Calc m/z	△ppm	Ion	Obs m/z	Calc m/z	△ppm
				y10	1116.4259	1116.4277	1.6123
b3	184.0721	184.0717	2.1731	y9	1059.4153	1059.4063	8.4953
b4	331.1402	331.1401	0.3020	y8	912.3373	912.3379	0.6577
b5	448.1649	448.1649	0.0000	y7	795.3112	795.3130	2.2633
b6	517.1859	517.1864	1.1601				
b7	604.2175	604.2184	1.4895	y5	639.2583	639.2590	1.0950
				y4	542.2061	542.2068	1.2910
				y3	395.1374	395.1384	2.5308
b10	951.3464	951.3488	2.5227	y2	292.1290	292.1292	0.6846
b11	1038.3806	1038.3808	0.1926	y1	205.0969	205.0972	1.4627

a) The amino acid sequence of archalan α (**1**) is presented with b and y ions marked as well as the location of the thioether ring. LC-MS/MS was used to fragment **1** to confirm the amino acid sequence by examining the fragmentation patterns. **c**) Major fragment ions of the doubly charged parent ion (*m/z* 621.7382) (**b**), are annotated with their b/y ion's identity, and the amino acid residues deduced from fragment ions are labelled in blue/red, respectively. **d)** The observed and calculated b/y ions are listed in the table.

Supplementary Figure 12: Chemical structure of archalan α (1) with key ^{1}H-^{1}H COSY and ^{1}H-^{13}C HMBC correlations

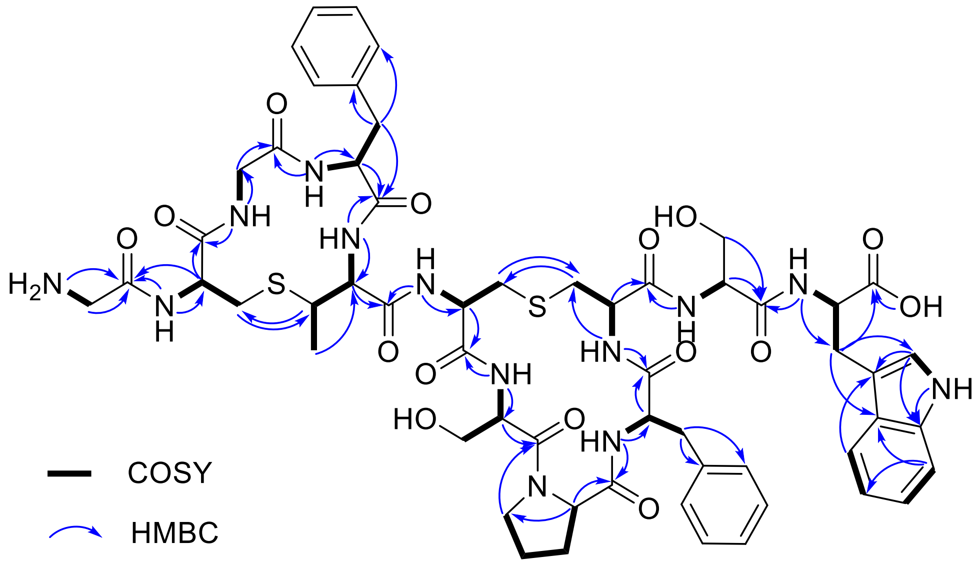

The structure of **1** was fully elucidated by NMR spectroscopy. Many exchangeable amide NH protons (δ_H 7.8-10.8 ppm) occurred in the ^{1}H-NMR and carbonyl carbons (δ_C 165-173 ppm) in ^{13}C-NMR spectra. Through HSQC, COSY and HMBC, the deduced structure included 2 × Gly, 2 × Phe, 2 × Ser, 1 × Pro, 1 × Trp, 1 × lanthionine (Lan), and 1 × methyllanthionine (MeLan). The HMBC correlations within Lan/MeLan subunits were consistent with our prediction based on HRMS that Cys2 had a crosslink with Thr5 through C-S bond and the same as Cys6 and Ser10. Key ^{1}H-^{1}H COSY and ^{1}H-^{13}C HMBC correlations are listed here and shown in Supplementary Figs. 13-14.

Supplementary Figure 13: Key COSY (a), HSQC (b), and HMBC (c) correlations within methyllanthionine subunit of archalan α (1) in d_6-DMSO

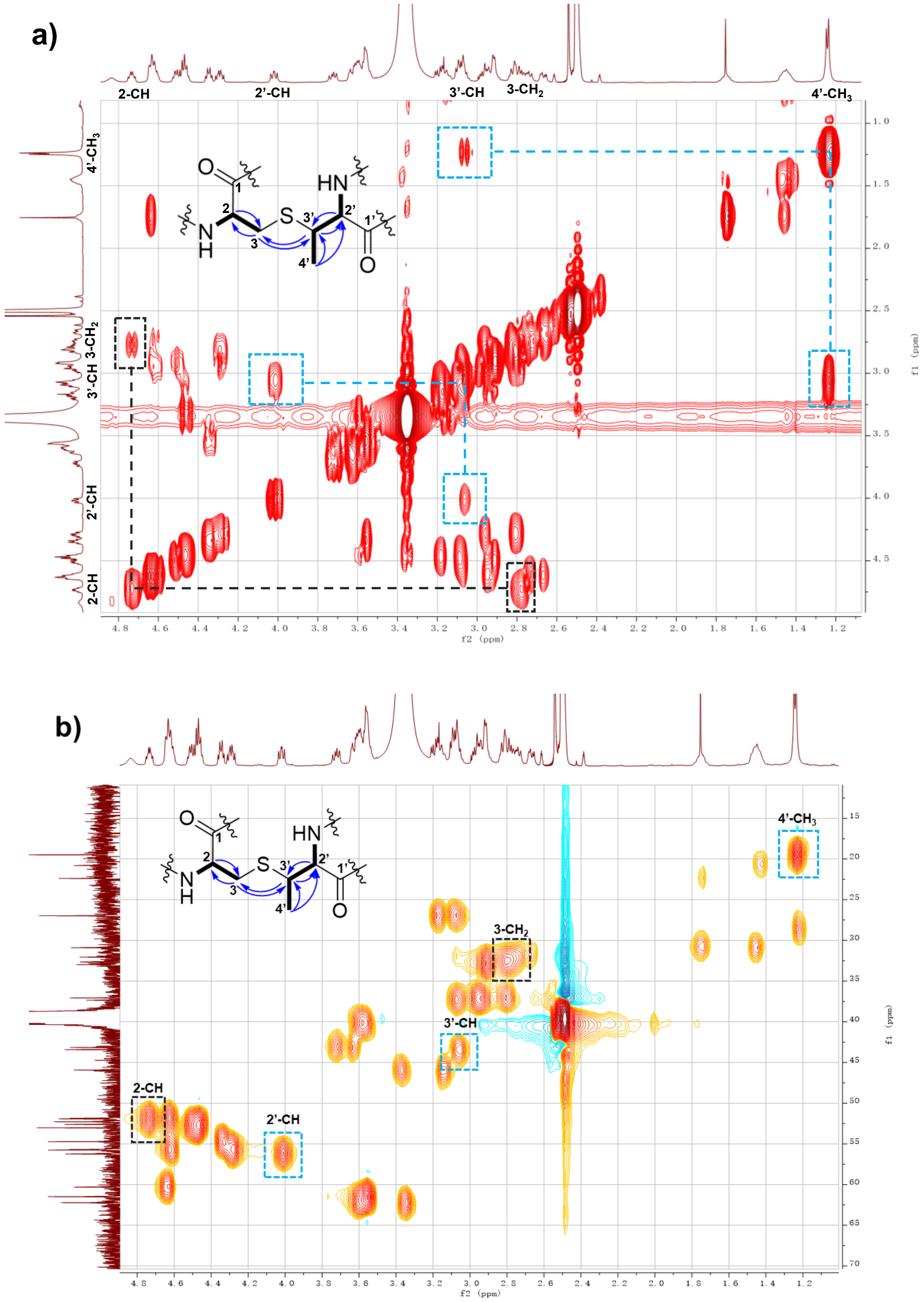

c)

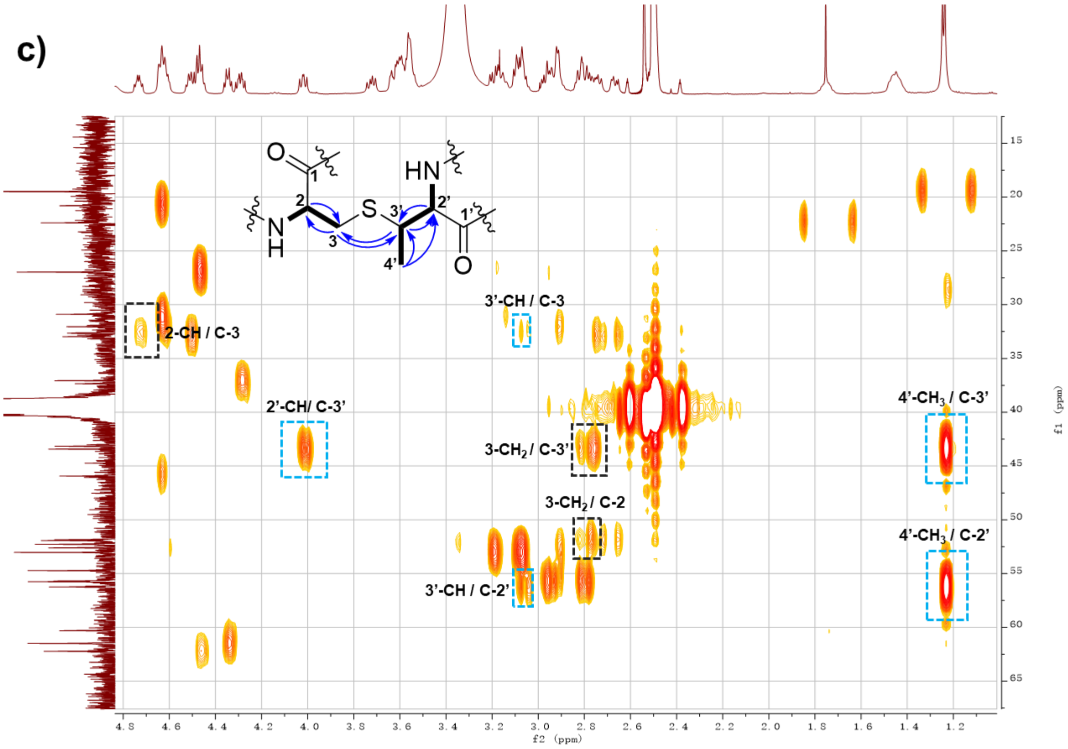
2-CH / C-3
2'-CH/ C-3'
3'-CH / C-3
3-CH$_2$ / C-3'
3-CH$_2$ / C-2
3'-CH / C-2'
4'-CH$_3$ / C-3'
4'-CH$_3$ / C-2'
f2 (ppm)
f1 (ppm)

Supplementary Figure 14: Key COSY (a), HSQC (b), and HMBC (c) correlations within lanthionine subunit of compound 1 in *d_6*-DMSO

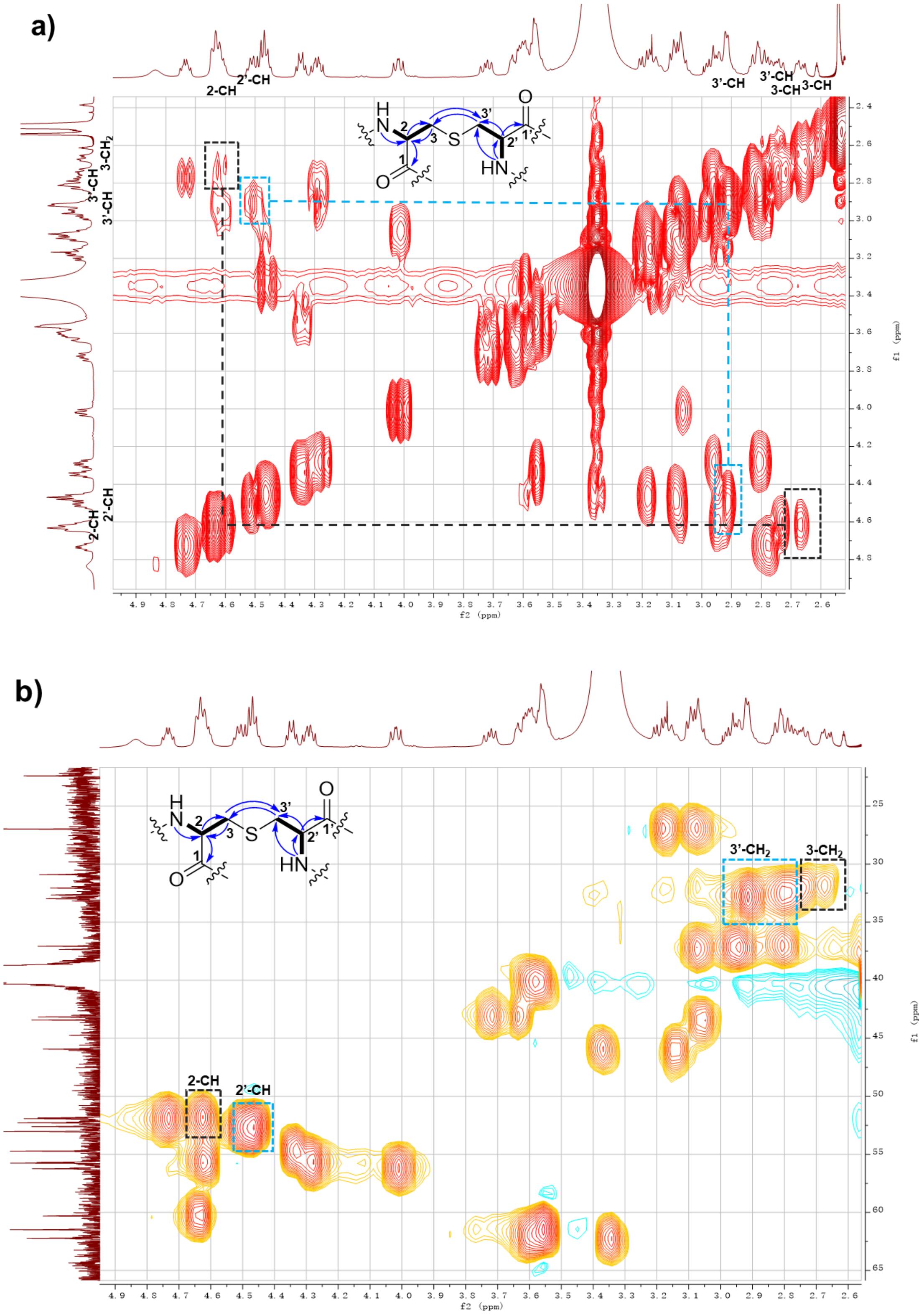

c)

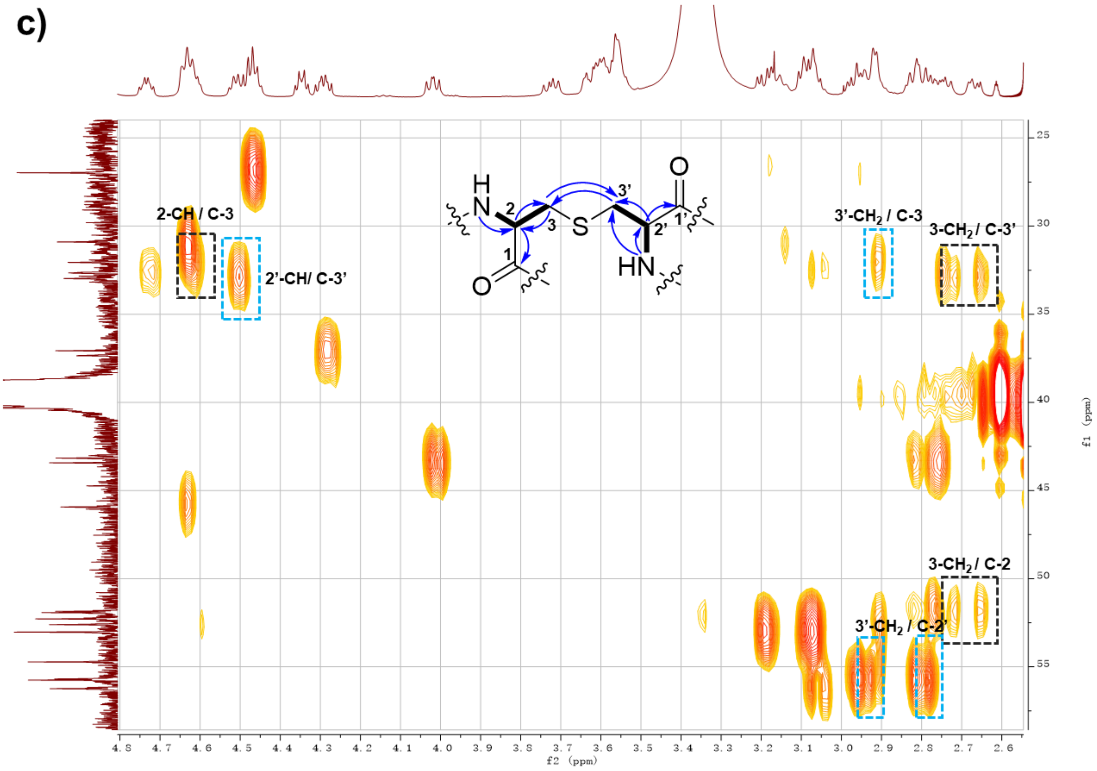

Supplementary Figure 15: Characterization of the C-S crosslinks in archalan β (2)

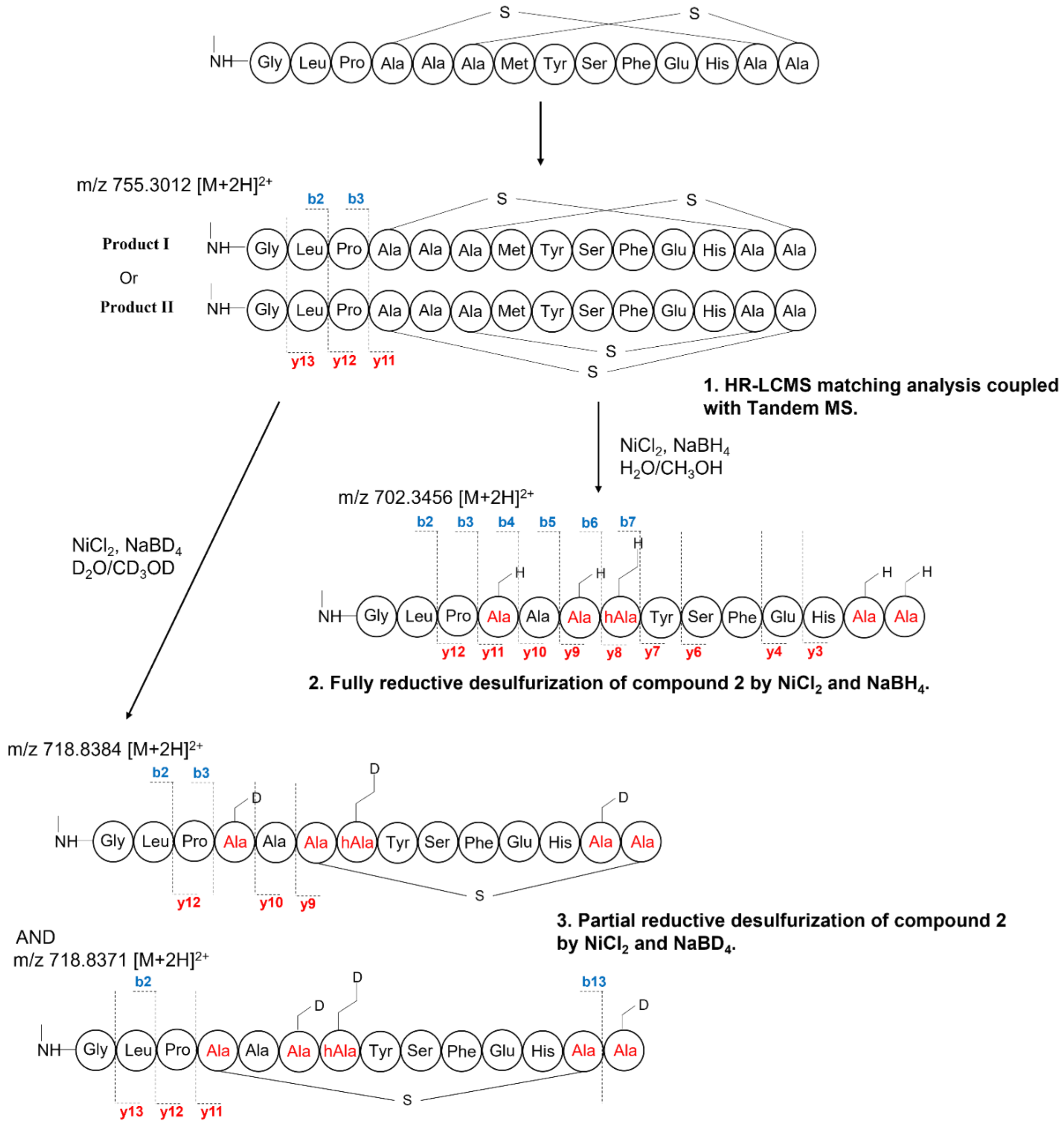

1) The tandem mass spectrum of archalan β (**2**) showed a linear GLP motif in the N terminal, consistent with the predicted core peptide sequence of *alnβ* (Supplementary Table 3, GLPSASMYSFEHCC). 2) To confirm the amino acid sequences, **2** was reduced and desulfurized by $NiCl_2$ and $NaBH_4$ treatment in H_2O/MeOH (1:1, v/v) to linearize. A tandem MS analysis of the full reduction-desulfurization product (702.3456 $[M+2H]^{2+}$) of **2** revealed the presence of four reduced Ala residues at the 4th, 6th, 13th, and 14th positions, supporting two C-S crosslinks between them, either as Ser4/Cys13 and Ser6/Cys14 intertwined topology or Ser4/Cys14 and Ser6/Cys13 hairpin topology. 3) To determine the ring topology, $NaBD_4$ and D_2O/CD_3OD were employed to desulfurize this peptide partially. Mass signals at m/z 718.8384 $[M+2H]^{2+}$ and m/z 718.8371 $[M+2H]^{2+}$ were identified in the partial desulfurized products, corresponding to two different partial desulfurized productions. Product I with m/z 718.8384 $[M+2H]^{2+}$ was the partial desulfurized product with an intact Thr6/Cys14 crosslink. Product II with m/z 718.8371 $[M+2H]^{2+}$ was identified to be the partial desulfurized products with an intact Ser4 and Cys13 crosslink. All the clues indicated that one ring formed between Ser4 and Cys13, and another was formed between Thr6 and Cys14.

Supplementary Figure 16: LC-MS/MS spectrum of archalan β (2)

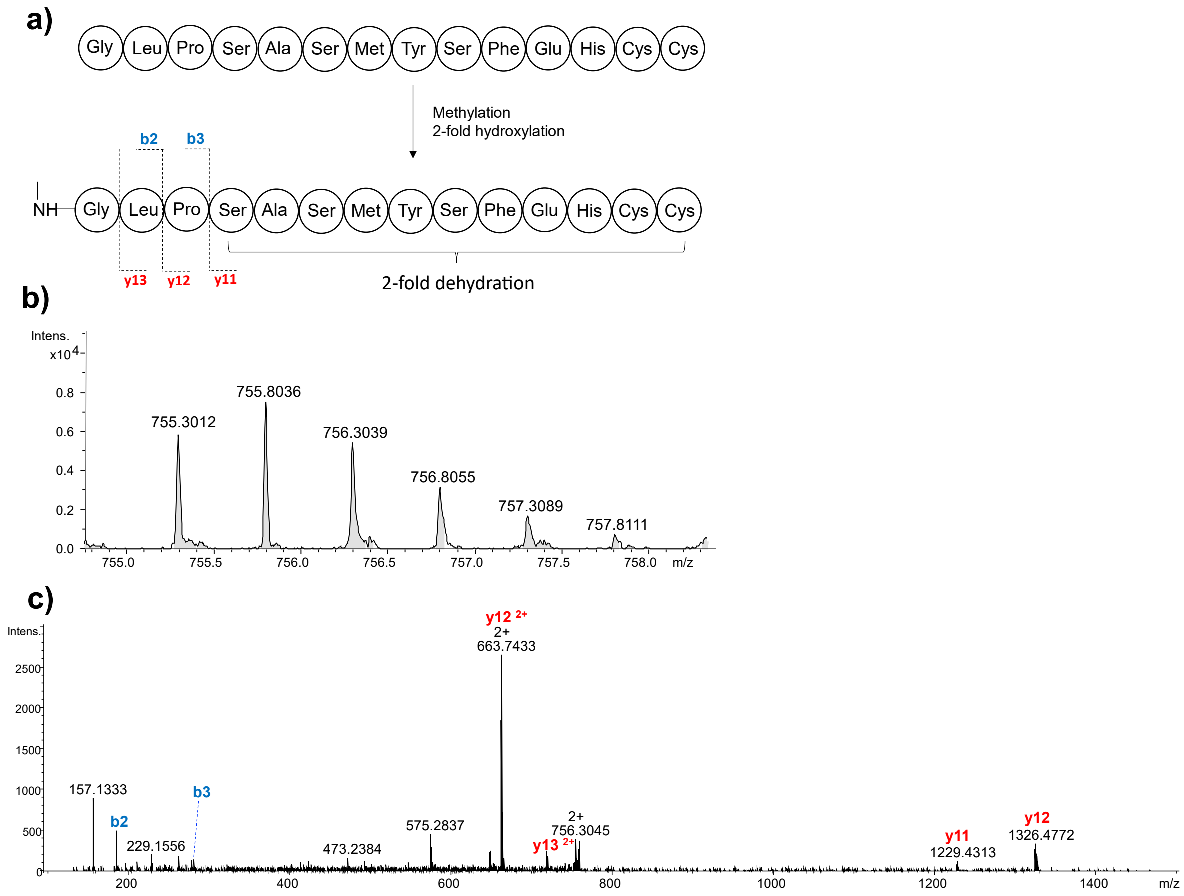

d)

Ion	Obs m/z	Calc m/z	△ppm	Ion	Obs m/z	Calc m/z	△ppm
				$y13^{+2}$	719.7854	719.7831	3.1954
b2	185.1280	185.1285	2.7008	y12	1325.4748	1325.4722	1.9616
b3	282.1829	282.1812	6.0245	y11	1228.4127	1228.4220	7.5707

a) The amino acid sequence of archalan β (**2**) is presented with b and y ions marked. LC-MS/MS was used to fragment compound **2** to confirm the amino acid sequence by examining the fragmentation patterns. **c**) Major fragment ions of the doubly charged parent ion, *m/z* 755.3012 (**b**), are annotated with their b/y ion identity, and the amino acid residues deduced from fragment ions are labelled in blue/red. **d)** The observed and calculated b/y ions are listed in the table.

Supplementary Figure 17: LC-MS/MS spectrum of fully desulfurized archalan β (2)

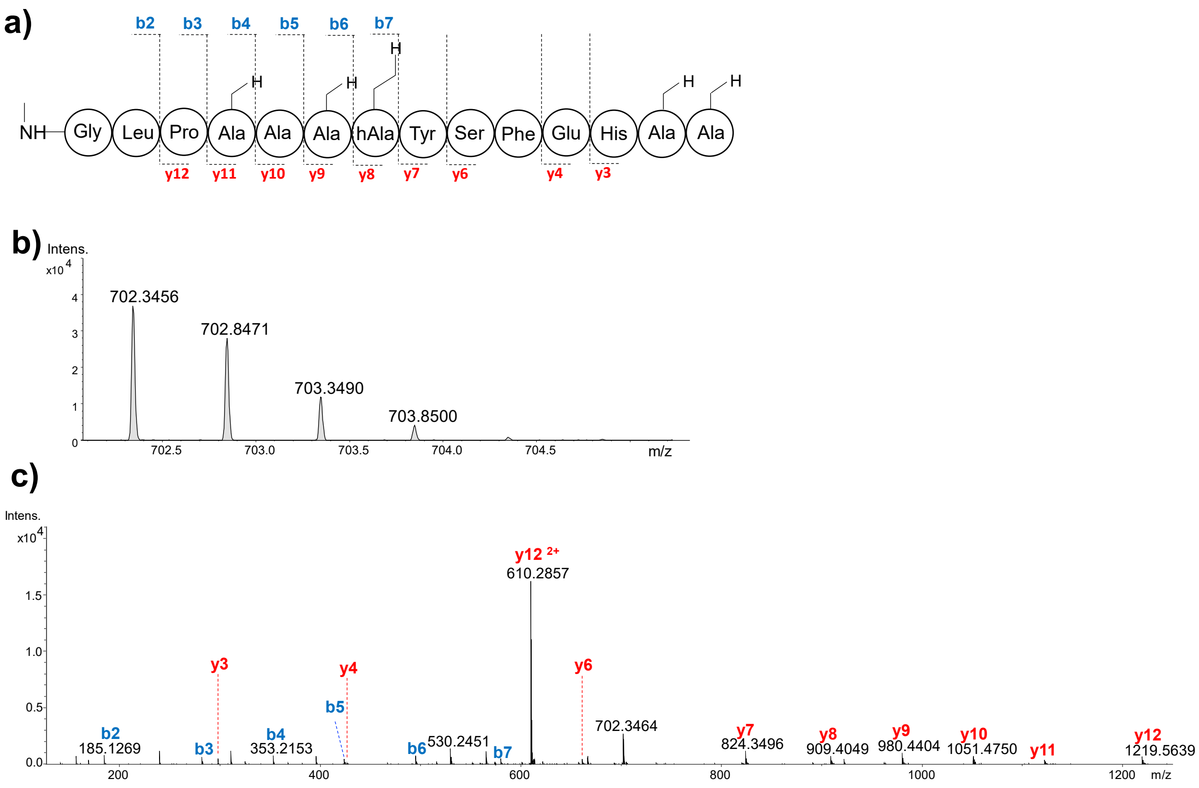

d)

Ion	Obs m/z	Calc m/z	△ppm	Ion	Obs m/z	Calc m/z	△ppm
b2	185.1269	185.1285	8.6426	y12	1219.5639	1219.5742	8.4456
b3	282.1778	282.1812	12.0490	y11	1122.5122	1122.5214	8.1958
b4	353.2153	353.2183	8.4933	y10	1051.4750	1051.4843	8.8446
b5	424.2537	424.2554	4.0070	y9	980.4404	980.4472	6.9356
b6	495.2908	495.2926	3.6342	y8	909.4049	909.4101	5.7180
b7	580.3418	580.3453	6.0309	y7	824.3496	824.3573	9.3406
				y6	661.2864	661.2940	11.4926
				y4	427.1891	427.1936	10.5339
				y3	298.1474	298.1510	12.0744

a) The amino acid sequence of the fully desulfurized product of **2** is presented with b and y ions marked. Compound **2** was reduced by $NiCl_2$ and $NaBH_4$ in H_2O/MeOH (1:1, v:v), generating a fully-desulfurized product with the double-charged parent ion at *m/z* 702.3456 (**b**). **c)** Major fragment ions of the fully desulfurized product of **2**, which are annotated with their b/y ions' identity, and the amino acid residues deduced from fragment ions are labelled in blue/red. **d)** The observed and calculated b/y ions are listed in the table.

Supplementary Figure 18: LC-MS/MS spectrum of partial desulfurized archalan β (2) product I

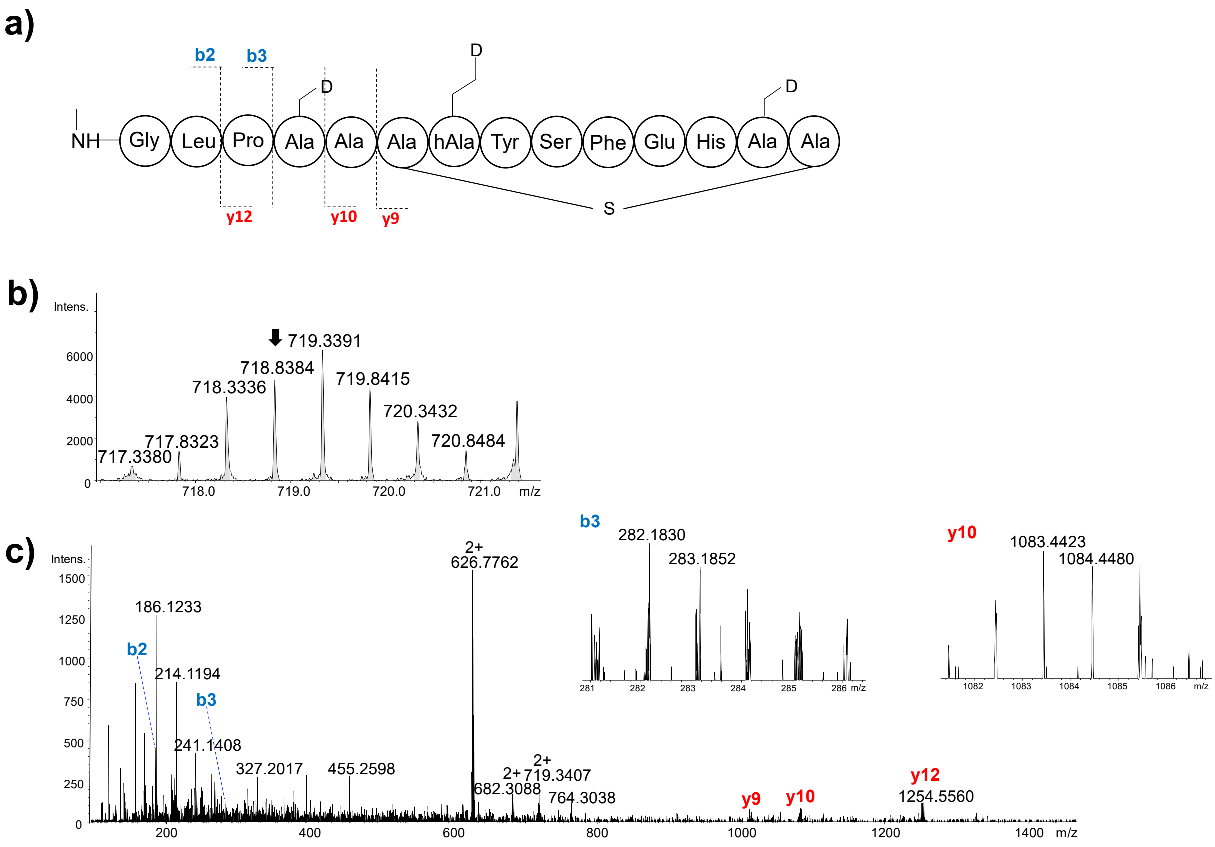

d)

Ion	Obs m/z	Calc m/z	△ppm	Ion	Obs m/z	Calc m/z	△ppm
b2	185.1276	185.1285	4.8615	y12	1253.5553	1253.5567	1.1168
b3	282.183	282.1812	6.3789				
				y10	1083.4423	1083.4533	10.1527
				y9	1012.4119	1012.4162	4.2473

a) The amino acid sequence of partial desulfurized product I of **2** is present with b and y ions marked and the location of the thioether ring, which was generated from partial reductive desulfurization by $NiCl_2$ and $NaBD_4$ in D_2O/CD_3OD (1:1, v:v). LC-MS/MS was used to fragment the partial desulfurized product I to confirm the ring position by examining the fragmentation patterns. **c**) Major fragment ions of the doubly charged parent ion at *m/z* 718.8384 (**b**), are annotated with their b/y ions' identity and the amino acid residues deduced from fragment ions are labelled in blue/red, respectively. **d)** The observed and calculated b/y ions are listed in the table.

Supplementary Figure 19: LC-MS/MS spectrum of partial desulfurized archalan β (2) product II

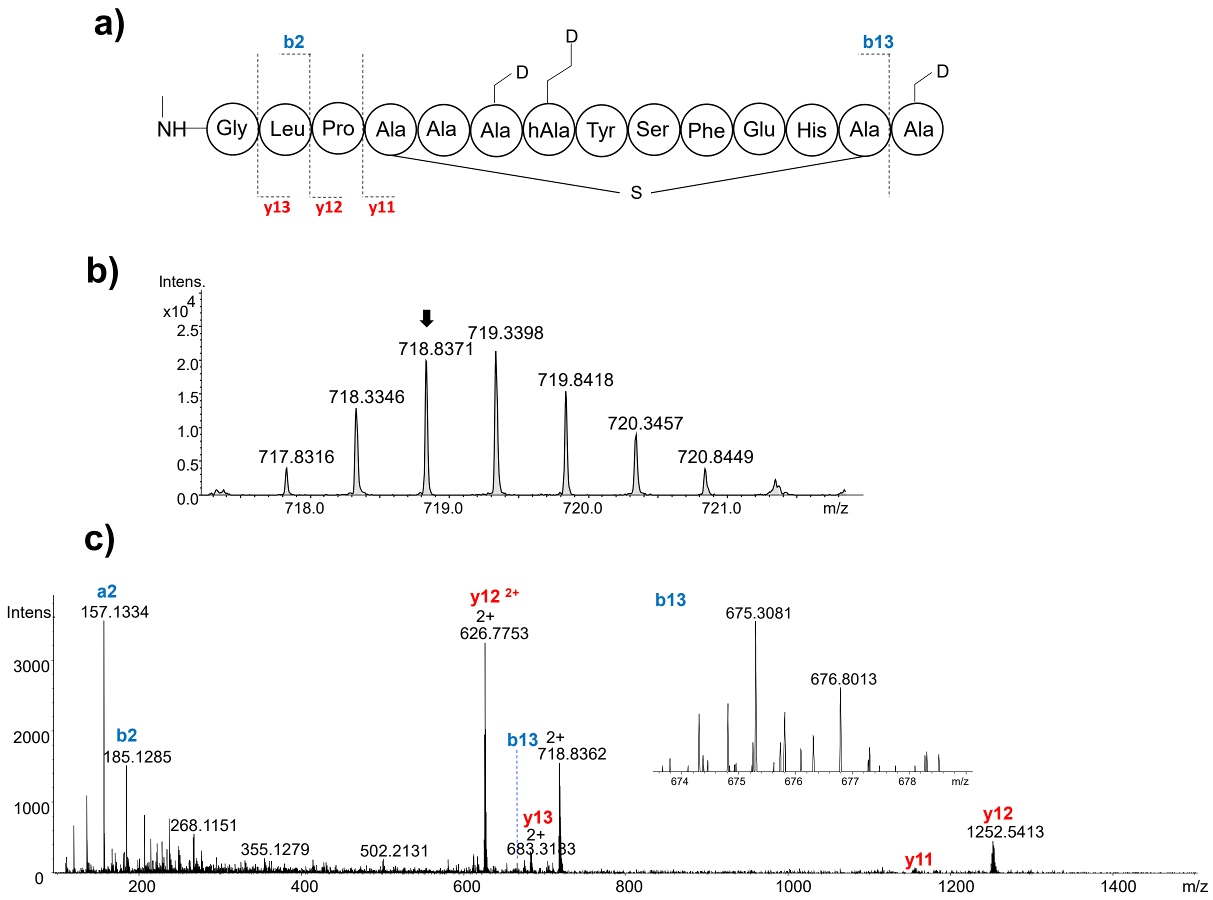

d)

Ion	Obs m/z	Calc m/z	△ppm	Ion	Obs m/z	Calc m/z	△ppm
				y13 $^{+2}$	682.8126	682.8165	5.7116
b2	185.1276	185.1285	4.8615	y12	1253.5553	1253.5567	1.1168
				y11	1155.4817	1155.4967	12.9814
b13	675.3081	675.3230	22.0635				

a) The amino acid sequence of partial desulfurized product II of **2** is presented with b and y ions marked as well as the location of the thioether ring, which was generated from partial reductive desulfurization by $NiCl_2$ and $NaBD_4$ in D_2O/CD_3OD (1:1, v:v). LC-MS/MS was used to fragment the partial desulfurized product II to confirm the ring position by examining the fragmentation patterns. **c**) Major fragment ions of the doubly charged parent ion at *m/z* 718.8371 (**b**) are annotated with their b/y ions' identity and the amino acid residues deduced from fragment ions are labelled in blue/red. **d)** The observed and calculated b/y ions are listed in the table.

Supplementary Figure 20: Structure feature of class II lanthipeptide

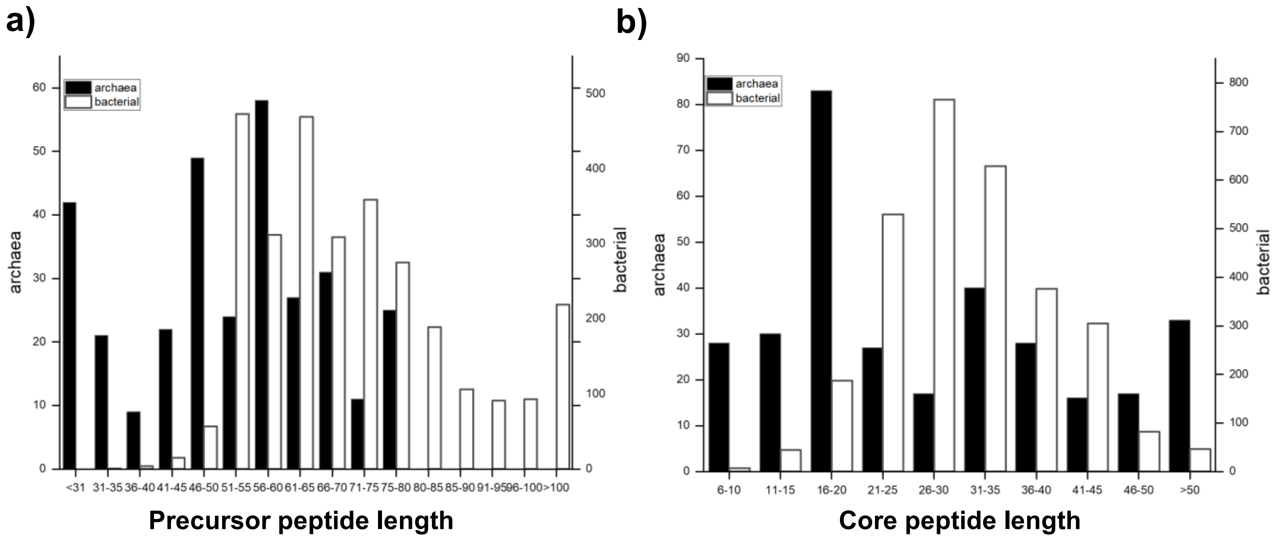

a) Length distribution of bacterial/archaeal class II lanthipeptide putative precursors. Generally, the length of archaeal precursors is shorter than bacterial precursors. Precursors from both have a dominant number in the length of 51-70. **b**) Length distribution of bacterial/archaeal class II lanthipeptide putative core peptides. Most of the archaeal core peptides were distributed in 16-20 amino acids, shorter than bacterial ones (21-35).

Supplementary Figure 21: Topology of bacterial class II lanthipeptide

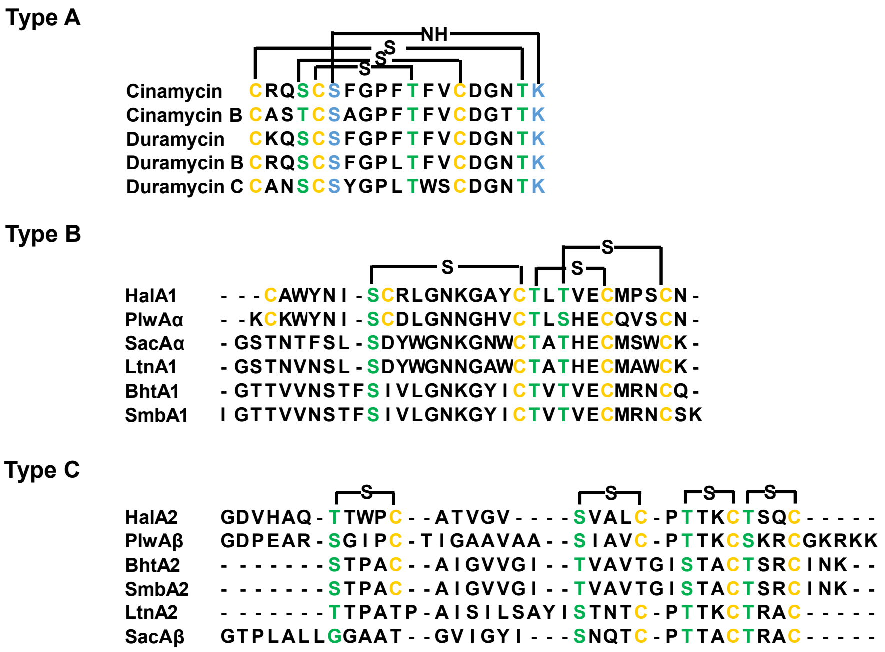

There are three typical topologies of known class II lanthipeptides of bacterial origin: hairpin, intertwined, and bicycle. Type A bacterial lanthipeptide (e.g., cinnamycins and duramycins) formed a hairpin topology. Type B lanthipeptide exhibit an intertwined topology, representing by HalA1, PlwAα and etc. Type C was represented by HalA2, PlwAβ, SacAβ, LtnA2, BhtA2 and SmbA2, forming non-overlapping 'bicycle' rings.

Supplementary Figure 22: Topology of archaeal class II lanthipeptide

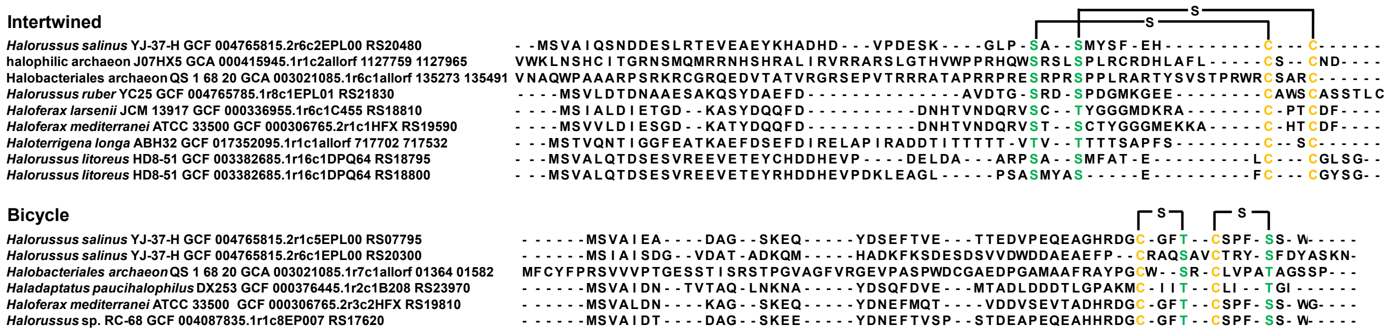

Based on the identified archaeal class II lanthipeptide and bioinformatically identified precursors, the topologies of some archaeal lanthipeptides were proposed to be intertwined and bicycle.

Supplementary Figure 23: NMR spectra of archalan α (1) in d_6-DMSO

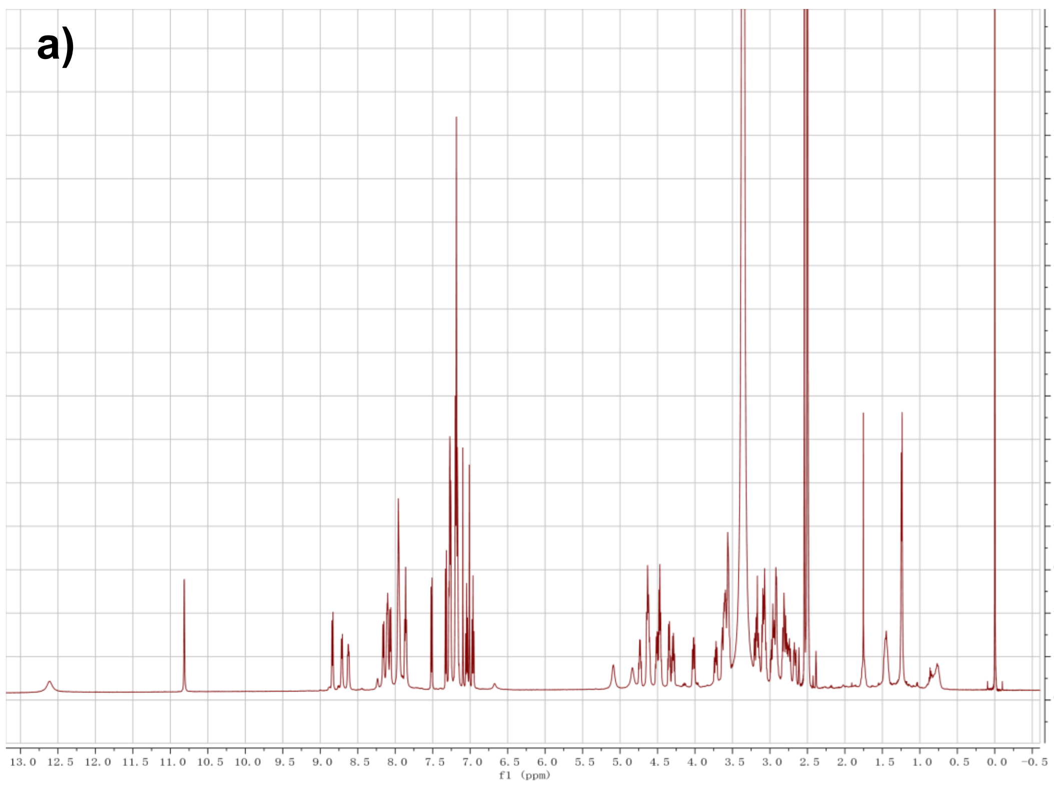

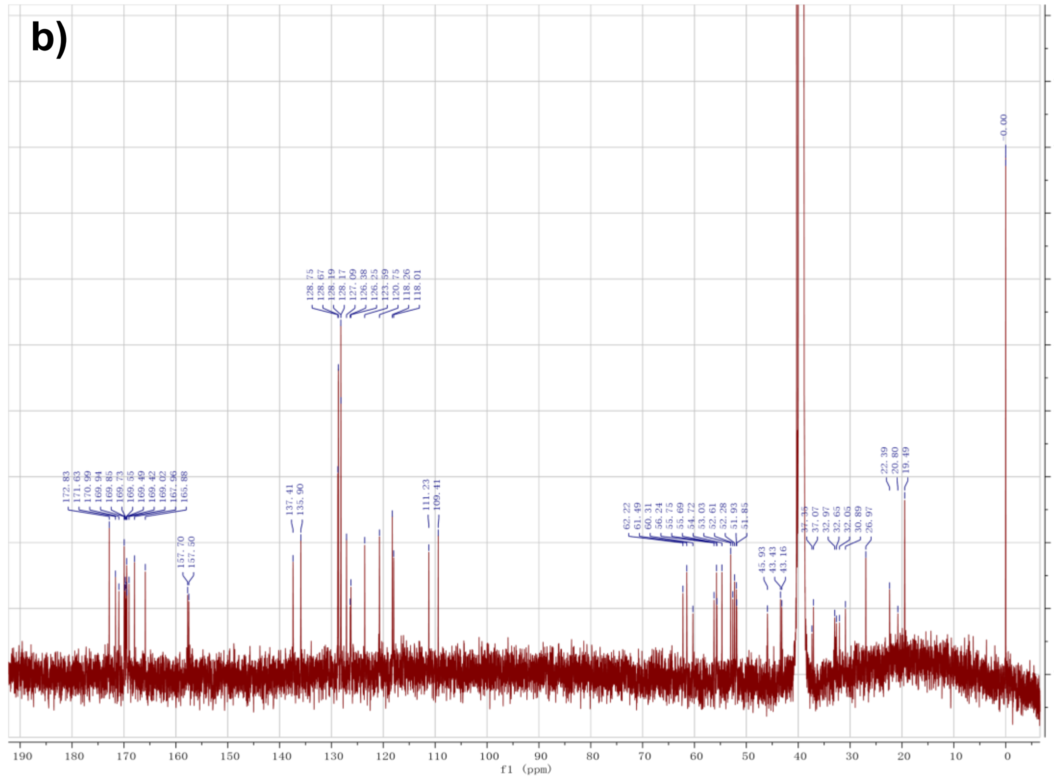

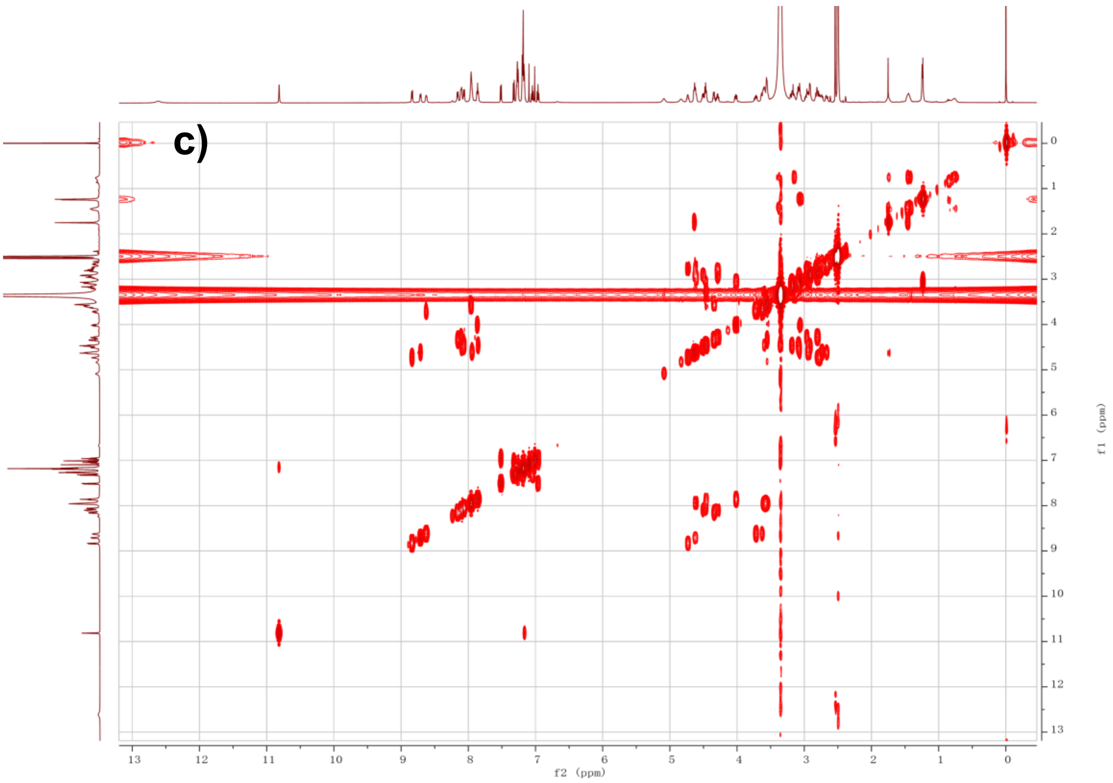
c)
f2 (ppm)
f1 (ppm)

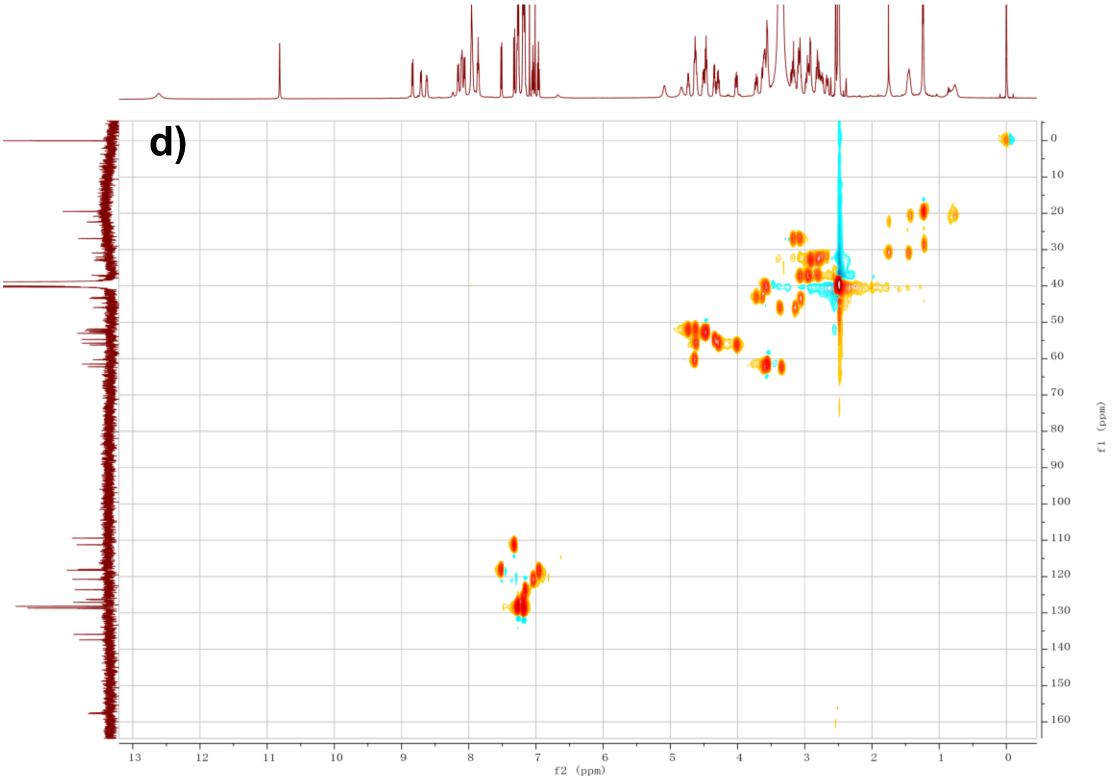
d)
f2 (ppm)
f1 (ppm)

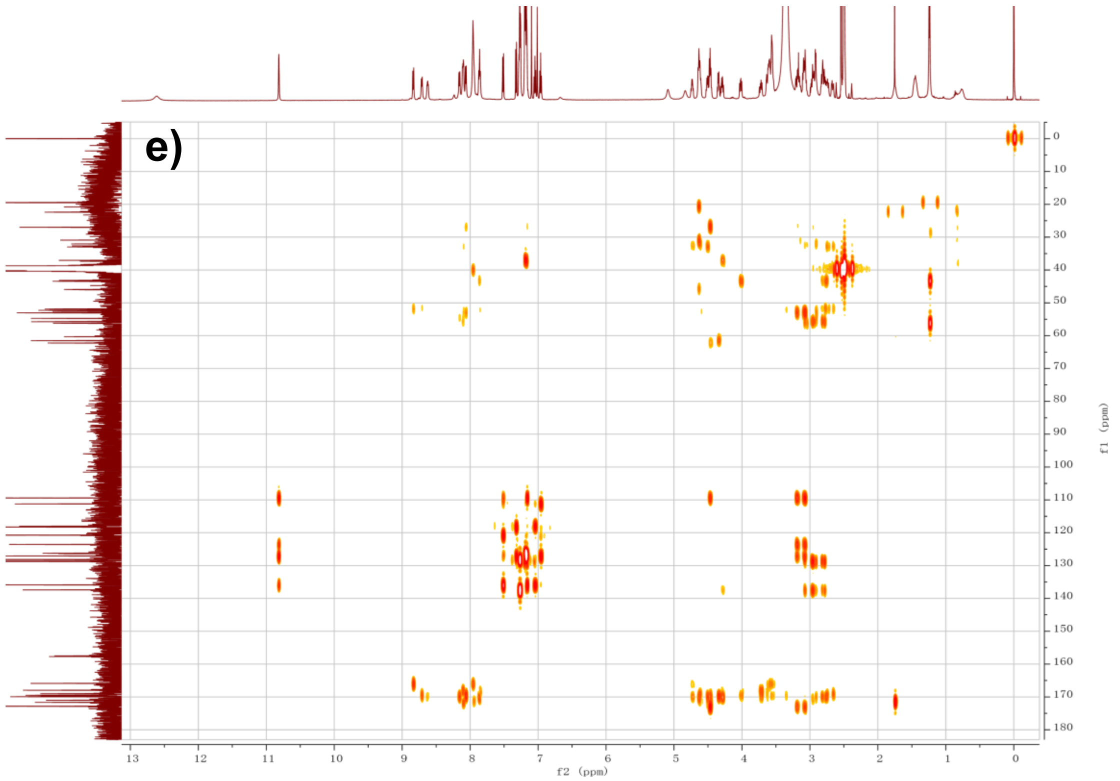

^{1}H-NMR (**a**), ^{13}C-NMR (**b**), COSY (**c**), HSQC (**d**), and HMBC (**e**) spectrum are shown in order.

Supplementary Tables

Supplementary Table 1. List of putative precursors identified in this study and previous studies

Peptides highlighted in yellow represent for known precursors discovered in both this study and previous studies by Walker *et al.* (2020) and Castro *et al.* (2021). Peptides in bold were new precursors detected in this study. Peptides and amino acids in red represent the differences between the LanAs identified in this study and previous studies.

Species (lanM)	LanA sequence (this study)	LanA sequence (previous studies)
*Haladaptatus_cibarius*_D43 (HL45_RS00765)	**MSVAIDNDTTCTVDKDAFDAEFEAIDAPEENELGHTRTATASCYLPCHTASKLPCCDTTN**	
	MNLVMSATSMPTNDLKAQFEDKYARDEPTIDELEHDAAAPDSCGFNSCNTQLQ	MSATSMPTNDLKAQFEDKYARDEPTIDELEHDAAAPDSCGFNSCNTQLQ
*Haladaptatus_cibarius*_D43 (HL45_RS12075)	**MSQPSTNSNTTPPHRTRVGSTVATRNFSKRAVVGASTVRCCISSIAVFSLCTILT**	
	**MVLTVLLVAGFAVALPVRVWLLATQTARLVAWATGTTIGSVEPAVLTRILRRRSKCVFGR**	
*Haladaptatus_cibarius*_D43 (HL45_RS19115)	**MACTCPHHVHRNAFCKHMAAVENAINDGTLDAFPSEDENGAEPDDCDCDGLGDFRCWPCVRTGRKELPN**	
	**MCECSMKLDITDESERGRNAPVSAGVAGSCSCSHTCTCCCTCSCNSA**	
*Haladaptatus_paucihalophilus*_DX253 (BUB61_RS13380, B208_RS0118315, ZOD2009_RS19365, SAMN05444342_2764)	**MSVVIENKSSIQFDKKKYDAEFDVPMETPDIDDDEFVAKADDSWCLITCGVFTCTITG**	LYCMSVVIENKSSIQFDKKKYDAEFDVPMETPDIDDDEFVAKADDSWCLITCGVFTCTITG
	**MSVAIDNTVTAQLNKNAYDSQFDVEMTADLDDDTLGPAKMCIITCLITGI**	
	**MSVAINSTAAQIDKESYDAEFDAATSETEFDEDDLLGPAEMCWFSCGLSCGLTG**	
	**MSIVIDSPVAAQLNKDVYDSEFKTDALVPEYEDEEDLGPAKLCLLTCTVSCAVTVSV**	
*Haladaptatus_paucihalophilus*_DX253 (ZOD2009_20422)	**MIENKSSIQFDKKKYDAEFDVPMETPDIDDDEFVAKADDSWCLITCGVFTCTITG**	
	**MTAQLNKNAYDSQFDVEMTADLDDDTLGPAKMCIITCLITGI**	
Haloarcula argentinensis DSM 12282		MNMESYSINSIGEQLDKDTYDAEFDEVTLSPESDNQDIPGVAKADDDKCWVLTCGITVFPSDE
*Haloarcula_salaria*_JCM_15759 (GCM10009006_36120)	**MNMESYSINSIGEQLDKDTYDAEFDGVTLSPESDNQDIPGVAKADDDKCWVLTCGITVFPSDE**	
*Haloarcula_salaria*_JCM_15759 (IEY10_RS18130)	**LSGIKKVVHSRRSARTGGSRSTRDQTERDGPVTKVLFQTPYCYVFSCVML**	
	**LNSSERSSWNRRIASSALIISRNRLKASEIMSRTAILSGPCSKRSRMYMANSGAIGW**	
*Haloarcula_salaria*_JCM_15759 (IEY10_RS18130, GCM10009006_36120)	**MSASAAACRRHDSVAALGVADPVSDYRSNRLSGGRLGETVHRNESVSVGGDRGCPYWMGYLHSDHTV**	
	**LFNNHFIGVLEENLYSVEGSCRNNFLPTILFHR**	
	**LSNFEWLRCRESSKFACMVGGSTRLGVWRAI**	
Halobacteriales QS 1 68 20 (BRC81_02815)		MSVVIDGRAAAQSSKKNTFDSEFDLAKFLPAFGEDTDQTAEADPCSWTCSWTCSWTSTY

Halobacteriales QS 1 68 20 (BRC81_03730)		MSVAIDAQAEPTSKDAFDAEYGEVSPESADEHEACLYDSAVCDTQTSNP
Halobacteriales QS 1 68 20 (BRC81_05635)*		MITDLVTVAEALAPRDTGLGLSYDKRLTSVSTAGSFRTYSNACEKTMTETDIVT EIADKHNQDLTPGTEEHTAGNCGGTIITGCF
Halobacteriales QS 1 68 20 (BRC81_06440)		MSAVIESSADGKAEFDNEFADAAYEKDDDADVVGAPCGCGTSGSSIASP
Halobacteriales QS 1 68 20 (BRC81_09010)*		MDYGSAELKRTFDDHFDTVDEAEVASKDSIYSISCTFLCSGGC
Halobacteriales QS 1 68 20 (BRC81_15050 and BRC81_15045)*		MSRKSNLKAELESRYERTEVEIPGEETGCSFSEDHCPDCSTY<br>MSRKSNLKAELESRYERTDVDIPDNEAGCSFSESICPDCSIA
Halobacteriales QS 1 68 20 (BRC81_15310)		MSTNGTRDLKAQFEGKYARAEPTITQSTPKDSCGSESCNDGVSE
Halobacteriales_archaeon_QS_1_68_20 (BRC81_02815)	**VNAQWPAAARPSRKRCGRQEDVTATVRGRSEPVTRRRATAPRRPRES RPRSPPLRARTYSVSTPRWRCSARC**	
Halobacteriales_archaeon_QS_1_68_20 (BRC81_03730, BRC81_03735)	**VTRMGVGGLRVTDRRVVQARPVFVGGLRRALTVFGVERVVARRFGL RVDCDLHQTAY**<br>**MFCYFPRSVVVPTGESSTISRSTPGVAGFVRGEVPASPWDCGAEDPGA MAAFRAYPGCWSRCLVPATAGSSP**	
Halobacteriales_archaeon_QS_1_68_20 (BRC81_06440)	**MEYESKVNDIFEGEDAIALCLYNRAEFPSSGICDVVRVHP**<br>**VGRGRIGGPPGGYVGAPRRGRPPRPVRSTATGRRRPRRRWRHAATR RSPADRVTRGSRAGSRTGGPRSPCRWPT**	
Halobacteriales_archaeon_QS_1_68_20 (BRC81_13345)	**VFECLLRRRSGERHGGSCSRSGRSRCSSRSAGSPSHGRGCAAVGRRTR CRCPGS**	
Halobacteriales_archaeon_QS_1_68_20 (BRC81_15310)	**VQKHVSLHTSGTVWTATGSRRMVGRRIPLLFAHSVVTTLGPAGVLG RRLGDRRLRAGVLAFELCFQVASAICRHGKS**	
Halobiforma lacisalsi AJ5_pHLAJ5I		MSVNEITFGMKYGTSGRIDERIRSIFDEKFETNYLSILGLTITPGPCSTGAPTLLCP DTCSCGCSITCEGSTYGCDC
*Halobiforma_lacisalsi*_AJ5 (C445_16764)	**MTSTQSLTTRRSSVTLRRVSSRHLPTKIALTNWILSTSVGRLITTFGTT VCSPWTGETRLSRR**<br>**LRGHSSLLFWSIAGAVHRGTTGSALDQRTSPAEAGVSPLIPLLSIASTG SCTAC**	
Halobiforma_*lacisalsi*_AJ5 (C445_*RS16495*, CHINAEXTREME_RS20780)	**LLIFYRVIKLVEFGRVAAALNCILRRTSRSRN**	
*Halobiforma_lacisalsi*_AJ5 (C445_RS16495, CHINAEXTREME_RS20780, C445_16764)	**VMSATQQSFGMSYDVTDTSLKTAFDERFGAEDVTDLPLSLTIDWSCSC GCSIGCNCNSCGCGC**	
*Halobiforma_lacisalsi*_AJ5 (C445_RS16495, CHINAEXTREME_RS20780, C445_16764, CHINAEXTREME_20785)	**MVRVGSIHGTVRDFRPVIPRYPTCVRAVQCAVITDDSASCSDTNSLVV HSG**<br>**MSGIQSSFGMTYNERDDALRAIFDERFGAFPSKEAGPIISNGCTGSCLC TVFDCDCEPSCNECTG**<br>**MSTQQASFGMAHKQRDSELKVIFDDQFENWTKKAGIASLDCTGSCLC SLSCDCETCGGEC**	

	**MSTQQASFGMAHKQRDSELKAIFDDQFENWTKKAGIATLDCTGSCL CSLSCDCETCGGECN**<br>**VRRVHSGEDSSRRYRPRRPDADRATTHDRQAVGMKSVETVFVACSS**	
*Halobiforma_lacisalsi*_AJ5 (C445_RS16495, CHINAEXTREME_RS20780, CHINAEXTREME_20785)	**VPSLSANTRLYWSVTWWPQLSQRRFGKASQFASEIFGNCGGVDTGVY**	
*Halobiforma_lacisalsi*_AJ5 (CHINAEXTREME_20785)	**MSATQQSFGMSYDVTDTSLKTAFDERFGAEDVTDLPLSLTIDWSCSC GCSIGCNCNSCGCGC**	
Haloferax alexandrinus Arc-Hr		LMSVALDIGIARTVDKSHYDGLFGSIISDKFEMSHDSTGTASCYLPCHTASKLPC CDTTN
Haloferax denitrificans ATCC 35960		MGSVMVTSSNIAASERAKSTAASPTAPAATASVSVVSSLTSVDSVAPAIIRHLR ASGACAPRCTATRC
Haloferax mediterranei ATCC 33500		VGGILTHMSVVLDIESGDKATYDQQFDDNHTVNDQRVSTCTYGGGMEKKAC HTCDF
Haloferax sp. Atlit-4N (C5C07_19040)		MLSMFRFLDRCREVCHSGVEFFTHCYQVIQAIALLPSCSACFASSTDAVRSAIPA SNSSLIFIHRRDFLVQSHVLITTL
*Haloferax_denitrificans*_ATCC_ 35960 (C438_03732)	**VPPPSTVSRSATTAPTDSSRWSTGTSPAGSTASTRWTPSARRARSCSAS TPRPAS**	
*Haloferax_denitrificans*_ATCC_ 35960 (C438_RS03580, C438_03732)	**MSVALDINIARTVDKSHYDGLFGSIISDKFEMSHDSTGTASCYLPCHTA SKLPCCDTTN**	LMSVALDINIARTVDKSHYDGLFGSIISDKFEMSHDSTGTASCYLPCHTASKLPC CDTTN
*Haloferax_gibbonsii*_ARA6 (ABY42_RS17125, ABY42_17180)	**VDVIVRARTSTHDLLALGGVSAGRRVGRFQTDESESFVWFEIWEDAL CGRNKTVASRSCRSCLVANNLL**	
*Haloferax_larsenii*_JCM_13917 (C455_RS00665, C455_00672)	**LYSYCRNIVSSHGIRGVLSATYANYSHRLVGGCPTTFGTTLHIYPECY**<br>**MSIALDIETGDKASYDQQFDDNHTVNDQRVSTCTYGGGMDKRACPT CDF**	
*Haloferax_mediterranei*_ATCC_ 33500 (*BM92*_04890)	**VPSPSPASLASTTGSPSTPAGCTRSRSVSGTSGSR**	
*Haloferax_mediterranei*_ATCC_ 33500 (C439_00640)	**LETSTAGCFERDFESSPPPRSPYSLSYCEHILADNCSHYIKNTRRMRIRI VELRSASNRGLSLEYSDQTVLEKRRASNHCCPLSCQLKGRPKWQE VVRRDFGHVVDSLHEFVVVLLFATSLVIKCN**	
*Haloferax_mediterranei*_ATCC_ 33500 (E6P09_17495, BM92_18430, BM92_18450, C439_00640, C439_00650, HFX_5112, HFX_5116)	**MLSRITFAMSVALDNKAGSKEQYDNEFMQTVDDVSEVTADHRDGCG FTCSPFSSWG**	
Haloferax_mediterranei_ATCC_ 33500 (E6P09_RS07370, BM92_RS04680, C439_RS06040, HFX_RS04210)	**MSVVLDIESGDKATYDQQFDDNHTVNDQRVSTCTYGGGMEKKACHT CDF**	
*Haloferax_mediterranei*_ATCC_ 33500 (E6P09_RS07370, BM92_RS04680, C439_RS06040, HFX_RS04210,	**MSSVRSRPHEFTFFDHLFFVSTLAFAQFVLRVKLSHQRWRGHPCGSS RRV**<br>**MADEFTDKTTFGALKGLKQPTSQSFPFNHWCKIVLHECIFV**	

E6P09_07370, BM92_04890, C439_06130, HFX_0869)	
*Haloferax_mediterranei*_ATCC_33500 (E6P09_RS17495, BM92_RS16040, BM92_RS16055, C439_RS00605, C439_RS00615, HFX_RS15990, HFX_RS16005, E6P09_17495, BM92_18430, BM92_18450, C439_00640, C439_00650, HFX_5112, HFX_5116)	**MGRETLQRGNPICTLSTAITLLYTVVSVL**<br>**LQVIAHSTDRCRALGCVTSCKRYYNLSSSISSRPHIDVSSSSELNYSVSSTCLNRDSIVGVGPRAHCPSSHSLVYHG**
*Haloferax_mediterranei*_ATCC_33500 (E6P09_RS17495, BM92_RS16040, BM92_RS16055, C439_RS00605, C439_RS00615, HFX_RS15990, HFX_RS16005, E6P09_17495, BM92_18430, BM92_18450, C439_00640, C439_00650, HFX_5112)	**MYHQWGTVSGGCVACSVTSSD**
*Haloferax_mediterranei*_ATCC_33500 (E6P09_RS17495, BM92_RS16040, BM92_RS16055, C439_RS00605, C439_RS00615, HFX_RS15990, HFX_RS16005, E6P09_17495, BM92_18430, BM92_18450, C439_00640, C439_00650)	**MIYSLMSAYQTSEFGMGHSNKDDVLKSQFDDLFEKFFEIEGVGIETTIFGSFSCDCSCGSCICITDTQ**
*Haloferax_mediterranei*_ATCC_33500 (E6P09_RS17495, BM92_RS16040, BM92_RS16055, C439_RS00605, C439_RS00615, HFX_RS15990, HFX_RS16005)	**MSVALDNKAGSKEQYDNEFMQTVDDVSEVTADHRDGCGFTCSPFSSWG**
*Haloferax_mediterranei*_ATCC_33500 (E6P09_RS17495, HFX_RS15990, HFX_RS16005, E6P09_17495, HFX_5116)	**MIVVYYNNARTDLETGNSHRGFVPMAAVWCALRPTALELLCDLTTSEDELVRAFCSEPTTTQ**
*Haloferax_mediterranei*_ATCC_33500 (E6P09_RS17495, HFX_RS16005, E6P09_17495, HFX_5116)	**LQLVTYPIARQRSSECAMTCKHYYRCKSRIAPFSYRLSYDSDLPSWCV**<br>**MEWGRNLGGLILSRAFVLRCFLVIQLLSRSLRILARNKNSKCSLNTEIQN**
*Haloferax_mediterranei*_ATCC_33500 (HFX_5112, HFX_5116)	**MSPPCPLLSIRDADTGPARAVAGERPEYRRFDSDPFDFEELLEEIVKLAFKDIVLVTVPHSEFRCLIC**

	**MSAYQTSEFGMGHSNKDDVLKSQFDDLFEKFFEIEGVGIETTIFGSFSCDCSCGSCICITDTQ**	
*Haloferax_mediterranei*_ATCC_33500_CGMCC_1.2087 (HFX_5116)	**VELIQQLHSFTGSPRNTISRKRCFWLISSVIGLNTLD**	
*Haloferax_prahovense*_DSM_18310 (C457_RS08840, C457_09019)	**MSVTLDIGIARTVDKSHYDGLFGSIISDKFEMSHDSTGTASCYLPCHTASKLPCCDTTN**	LMSVTLDIGIARTVDKSHYDGLFGSIISDKFEMSHDSTGTASCYLPCHTASKLPCCDTTN
*Haloferax*_sp._ATB1 (ATB1_RS15910)	**MRSERRRPTVRLRRPQVVERRRHVAWSKSGLPLRGQAGGSAACPGRDGRSGSGVDYPNLPLM**	
*Haloferax*_sp._Atlit-12N (C5B90_RS16380, C5B90_16370), Haloferax_sp._ATB1 (ATB1_RS15910), *Haloferax_gibbonsii*_ARA6 (ABY42_RS17125, ABY42_17180)	**MSVALDIDIARTVDKSHYDGLFGSIISDKFEMSHDSTGTASCYLPCHTASKLPCCDTTN**	LMSVALDIDIARTVDKSHYDGLFGSIISDKFEMSHDSTGTASCYLPCHTASKLPCCDTTN
*Haloferax*_sp._Atlit-6N (DEQ92_RS16095, DEQ92_16095), *Haloferax*_sp._Atlit-4N (C5C07_RS17580, C5C07_17585), *Haloferax*_sp._Atlit-19N (C5B86_RS15300, C5B86_15290), *Haloferax*_sp._Atlit-16N (C5B87_RS15575, C5B87_15565), *Haloferax*_sp._Atlit-10N (C5B91_RS14745, C5B91_14735), *Haloferax*_prahovense_Arc-Hr (BN984_RS18090)	**MSVALDIGIARTVDKSHYDGLFGSIISDKFEMSHDSTGTASCYLPCHTASKLPCCDTTN**	
*Haloferax*_sp._Atlit-6N (DEQ92_RS16095, DEQ92_16095), *Haloferax*_sp._Atlit-4N (C5C07_RS17580, C5C07_17585)	**VDVIVRARTSTHDLLALGGVSAGRRVGRFQTDESESFVWFEIWEDALCGRNKTVVSRSCRSCLVANNLL**	
*Halomicroarcula_salina*_YGHS18 (GM656_RS00515), *Halomicroarcula*_salina_JCM_18369 (KTS37_RS04500, KTS37_04500)	**MEGFCGHTMARERSAETIPGLCAGVVTSRNVTGWIFQSVSYHYLVTCMWLV**	

*Halomicrobium_mukohataei*_DSM_12286 (Hmuk_0783)	**MAYSASNDLKTELDQLFDIDSPTIKAHDKKCFTESCTCSIACF**	
*Halomicrobium_mukohataei*_JP60 (E5139_07095)	**VRSIAVRGAKTRKKFFSDQRSPAVSSRNGTLCAPRDTVYCSCLPVIVA<br>TE**	
*Halomicrobium*_sp._ZPS1 (GBQ70_RS07090, GBQ70_07090), *Halomicrobium_mukohataei*_ZP12-2 (ELZ10_RS11700), *Halomicrobium_mukohataei*_JP60 (E5139_RS07080, E5139_07095), *Halomicrobium_mukohataei*_DSM_12286 (HMUK_RS03740, Hmuk_0783)	**LFAEPIACADQCSWRYRRSAPIGPRSA<br>VGLATHCSEGDSTLNVAQRPDTTDDERTTSTETIVNCVVILRSDPAVS<br>QPTTLLAL<br>MSERRTVSAGELAVEELLDRLESGERVVVRTEFLGSEHQVTLRYDGS<br>TFYCDTPTRLHRHESREAMRTCLENQGYASQ<br>LPPVTKGYCFGWRPPDGTPTACRFDGSAAATRCQTYSYGLL**	
Halophilic archaeon J07HX5 (J07HX5_01337)		MKADESDKKKYDAAFDRGDNTTARENMTADLAVPTCVLDSKMCMC
Halophilic_archaeon_J07HX5 (J07HX5_01230)	**MRPPANQTNKSPKSAVREAASFTTRRKSPLQQTTGTRGRKTPGSSLP<br>QSEHTHCLAP<br>LRRDAVELQTELVGCSDPSGENI<br>LNSTLGAWPRCSSHLRLDPRPVLTAILTRSQC<br>LDRDWAAALLPLVVEIIFSXXXXXXXXAACVRS<br>LSCWSSFRYASRQRPIYKLSDIETDQIAARLDTVIRFTLLRTLCASFLSI<br>GHTGVGIYLPRGSECNSGNLVTIVR<br>VSGSRTRASCFRDYCAGSAREHTGSAVSIGVDLAAPTPTRWSRQNVCP<br>GLSSPTNPDTHCTVTACWSVC<br>VWKLNSHCITGRNSMQMRRNHSHRALIRVRRARSLGTHVWPPRHQ<br>WSRSLSPLRCRDHLAFLCSRCND**	
Halopiger djelfimassiliensis IIH2*		MREQPSNTAVEQFETKHNRDVTEEFDESGPVAGCPIATCLVYSDN<br>MNEQSELTSVDQFEVKHNRDVNDVHEDGHQACYVLSCAVASGND
Halorussus sp. HD8-51		VTIGEKHSRMSATQTSTFGMTHSTTDSELKTQLDRLFESDAVPDTFDFGFSCSCI<br>CSFGCNGCSCCC<br>MNRTMFFSMSTNQTSFGMAYNAETEIKGVFDERFEEYTEKAAGVWSLSCTDS<br>CLCSGDCNGCSCGC
*Halorussus_litoreus*_HD8-51 (DPQ64_RS07015)	**MSTNQTSFGMAYNAETEIKGVFDERFEEYTEKAAGIWSLSCTDSCLCS<br>GDCNGCSCGC<br>MSTNQTSFGMAYNAETEIKGVFDERFEEYTEKAAGVWSLSCTDSCLC<br>SGDCNGCSCGC<br>MSTDQTSFGMTHEAENDIKGVFDERFEKYTGKMADIWSFSCTGSCLC<br>STGDCDTSNCY<br>MTHDQTSFGMAYNAETEIKGVFDERFEEYTEKIAGTWTLGCTDTCLC<br>STTVDCDPYANY<br>MSATQTSTFGMTHSTTDSELKTQLDRLFESDAVPDTFDFGFSCSCICSF<br>GCNGCSCCC<br>MSVTQTSTFGMQHSTSDSSLKQLFDEKFDVDLGDVHTVEWSGWCTG<br>SCLCTNIACDGCSCGC**	

*Halorussus_litoreus*_HD8-51 (DPQ64_RS11425)	**MRLQRIGVSSLASSRHYQSCLCHF<br>MSVALQTDSESVREEVETEYRHDDHEVPDKLEAGLPSASMYASEFCC<br>GYSG<br>MSVALQTDSESVREEVETEYCHDDHEVPDELDAARPSASMFATELCC<br>GLSG<br>VSERVAGNPRLVAVRRLIAMGGSSRASSATCLRSDTCWAHVSPDRSR<br>RRRSGSHSPRRRSLACVHLHYACYSLTY**
*Halorussus_ruber*_YC25 (EPL01_RS17120)	**MSVLDTDNAAESAKQSYDAEFDAVDTGSRDSPDGMKGEECAWSCASS<br>TLC**
*Halorussus_salinus*_YJ-37-H (EPL00_RS02450)	**MDESLLADWVRRSPSLDAILDSSARTAISDDFVPDDLLAEWRDVFDAD<br>ADFRDRLRFADWTPEETCDRPNPSEII<br>MPDEAAAIERCAYCRGYVPVTHVDIPDRPTDAPLRARVAICEKCLGD<br>GL<br>MARDISQSLESQYEPSHTETDDRAVIGETNCYLSTDPICGSSGEDDEME<br>F<br>VASRRTVVSSVVHAVRVALHAVITGVCGSPNRRGRRRGGNRSPCERR<br>GWVLSLDKIF**
*Halorussus_salinus*_YJ-37-H (EPL00_RS07375)	**VVGERCRILVHCPRPGGTRTSDGSRIQCQWLPVLATHRA<br>LSFWRMVFFSPPLLVICSFVNESFWCLIRPCCCPSSARVRVRNVNSS<br>MAVSDGPPIGIRHPTVNPTPDAAIAQSRSGACECVDCGYVVGTDDDLP<br>PKCPDCGGALTFVRS<br>MSVTQHTSDVKAEIETEYARDGSDALDDSSEPMRACSCYTRICCDIDS**
*Halorussus_salinus*_YJ-37-H (EPL00_RS07790)	**MSDFETYTCENCGDEFKAQESANAADESYCSPKCQTAA<br>MADNRVVQGRMVTPAALAEMIEGEDVMDAEAISETDRECPDCGGDV<br>LEVGYMPSVTEFVTGWKCQDCDWADTDRD<br>MSVAIEADAGSKEQYDSEFTVETTEDVPEQEAGHRDGCGFTCSPFSS<br>W**
*Halorussus_salinus*_YJ-37-H (EPL00_RS13700)	**LLREGTPVGGSCLCHRWCKARVV<br>VTATEGDGHLASLFGSRFDGSLVLFVCRFDLIF<br>MSSDSLKDQIEAAYEQDERPVEAGTEQAGQMTVAFCGGHYTNLE<br>VSEIGRLRISAWGSHLRCCRSRLTLRYYL**
*Halorussus_salinus*_YJ-37-H (EPL00_RS20295)	**MSIAISDGVDATADKQMHADKFKSDESDSVVDWDDAEAEFPCRAQSA<br>VCTRYSFDYASKN**
*Halorussus_salinus*_YJ-37-H (EPL00_RS20485)	**MSVAIQSNDDESLRTEVEAEYKHADHDVPDESKAGLPSASMYSFEHC<br>C<br>LIPAASLSADQPVAVALPTTAMTLETIIFPISIAAVGTNLDEGIQKSCKF<br>VE<br>LETIVFSSRHEGNYPPCALIVGGLAYR**
*Halorussus*_sp._HD8-83 (EPL03_RS06680)	**MSQDITASLEAEYEPEHDETDEQIVVGTSCPESLSVQPACMNSPDDEL<br>QF<br>MSRDVTTSLESEYEPARDETDRRAVVGSTGCHVSDKPLCESTGDATE<br>MEF**
*Halorussus*_sp._RC-68 (EP007_RS02715)	**LSKTEAFCNVANLRFATARKTRFVFRRATAPRQPREGRTTERGDGVK<br>RLGRCEARGCSAVPCGRGLSGVGSS<br>MSVKSDSFGTTHSSTTPDLKDEFEEDFTEQRQSKPLFRMASYTYCHSN<br>PVCTFCGPEDSDA**

	**LPLSLRSILHCLFIVYSRHYLTEIILKTREFSPLTACRIKLNG**	
	**VDSRLRKSVCTSVGESVVGGLASGICGVSSASRICEVDLVNRS**	
	**VRVRRRTCAVAPTARGMSERPNAVRGRANRLGRREADAAQCCAGRV<br>RYEWFKPVASFYSPNRPHRFCLPN**	
*Halorussus*_sp._RC-68<br>(EP007_RS04645)	**VVTVLLLTRARVAGCAGSMRGVTRVLTAACCAACERLCGDVWDWR<br>ESV**	
	**MNVSVWIGGHGTPADACARGGVCWLDEGRDTCPDCSVLRGV**	
	**LAETIRLADAHPSRSGEPAVPGREGGSLPACPFIRTLVRRSSRPAFSLLI<br>LC**	
	**VIWPVNESEEQFEIEIGVNTIQLGRKLIINNRDC**	
	MKRRTALKRLAAGAGSTFALVGAAGASDSEPKNLDDVEFKYRVTEDGLE<br>EVEPDDGFCEENPDCCVFDCSDCPYDCYDGCRCAG	LMWSFMKRRTALKRLAAGAGSTFALVGAAGASDSEPKNLDDVEFKYRVTED<br>GLEEVEPDDGFCEENPDCCVFDCSDCPYDCYDGCRCAG
	**MSRSITTSLEAEYEPSHDETDDRAVIGSTGCYISDKPVCIGSGKDDEME<br>F**	
*Halorussus*_sp._RC-68<br>(EP007_RS09960)	**MSRDSTASLESEYEPSHDETDDRAVIGSSICGASMDPICGSPGEDDEME<br>F**	
	**MVKTPDTDTPAAYSELTEECAECERETAHEVAVEIRTENPDSRASREP<br>YRVTECGVCGATEEVRLNNA**	
	**LIRMTELASDIPLTTMLVVCGTVLLLAGVPLGRDLITLAVGICVGESE<br>K**	
	**MSIAGVIASDLFFSLEKFHLPLQRGCNTVPKGALSCIPP**	
	**MSVAIDTDAGSKEEYDNEFTVSPSTDEAPEQEAHHRDGCGFTCSPFSS<br>W**	
*Haloterrigena_longa*_ABH32<br>(J0X27_RS03495,<br>J0X27_03495)	**VDAHSDTQSRGFPHLEDVHRGGPPNRERAESDSSASLSPWISTVGHRR<br>CHR**	
	**VAVPLASQRWHLRCPTVDIHGLRDADESLSALSRFGGPPRWTSSRCG<br>NPRD**	
	**VDSRTSRTSTVVGPRIERGQRATRRRLSARGYRLSDTVGAIAERREER<br>PPHPRTTNCRERSRQDTRLRAGGR**	
*Natrarchaeobaculum_aegyptiacu<br>m*_JW_NM-HA_15<br>(B1756_14695)	**VLRNTSAVLVQVRTGIVTERDRHVSRTIVAALNGGFFSFVHRFDALLS<br>LAVSRHCDF**	
*Natrarchaeobaculum_aegyptiacu<br>m*_JW_NM-HA_15<br>(B1756_RS14655,<br>B1756_14695)	**VMLRAPHHPARVCPDCETTLSNVQGLAACPRCSWVDRRDS**<br><br>**MARNSQAKKRIESMYEREEPTVEGGNDRAGHMTIAFCYDTSPDLH**	
*Natrinema_gari*_JCM_14663<br>(C486_RS10070)	**MSIGIDRDVENGATKDKHDADFDGEGAEELDTASMNTGTASCVLPCN<br>TASKHLPCNTASKHLPCCDTTS**	
*Natrinema*_sp._J7-2<br>(NJ7G_RS00200, NJ7G_0043),	**MSIGIDRDVENGATKDKYDADFDGEGAEELDTASMNTGTASCVLPCN<br>TASKHLPCNTASKHLPCCDTTS**	
*Natrinema*_sp._J7-1<br>(WQ9_RS14640)	**VAVYRTARLEATMLLLPVSGFVWLKRFANDATRRVLSRKTRFGAWP<br>QVPVCQSARRRRIQSSMT**	
Natronorubrum_tibetense_GA33<br>(C496_23276)	**VGDNRFRRDTGKQRRDNVHARRGRRGAPRVFPGCSGYACGKRPRGI<br>GHCADDDGTRARRVR**	
	**VIWFFLDLSHLQIENGGCILRFSRRCH**	

*Natronorubrum_tibetense*_GA33 (NATTI_RS0124325, C496_RS22820, C496_23276)	**LSGTVVGSNNVRKLRSHDREGEGAPLHGAPKRCHLSCIFLTDSLGQF GK**<br>**MGSTIPTPTDDKARETIGDATPGSLELDPTDDGFKAAMRQAIIDEELD DEALEVMTEISEAKYTFTCICTCKCTLSC**<br>**VWLDTTGHPAQNGTCRAGTSNESTSRTPSLRQRITTLADLQDVCASA FDHVDCARPISDSTTFGDGR**<br>**VSVRPTTPANNRNLASPSTSRGALDYITGSSGSGERC**<br>**VAAPQCPEGDERVEYGLGPCLREATQCVHGPSQ**	MESFKWRCMGSTIPTPTDDKARETIGDATPGSLELDPTDDGFKAAMRQAIIDEE LDDEALEVMTEISEAKYTFTCICTCKCTLSC

Supplementary Table 2. Predicted genes within the biosynthetic gene clusters of *alnα* and *alnβ*

Gene	*alnα* (description/protein ID)	*alnβ* (description/protein ID)
A	Hypothetical protein (WP_162224177.1)	Hypothetical protein (WP_162224287.1)
M	Type 2 lantipeptide synthetase LanM family protein (WP_135851040.1)	Type 2 lantipeptide synthetase LanM (WP_162224288.1)
T1	ABC transporter substrate-binding protein (WP_135851042.1)	
T2	DMT family transporter (WP_135852430.1)	
P	Matrixin family metalloprotease (WP_135851043.1)	
R1		ArsR family transcriptional regulator (WP_135854782.1)
R2		ArsR family transcriptional regulator (WP_135854781.1)
MT		4-Demethylwyosine synthase TYW1 (WP_135854783.1)

Supplementary Table 3. Observed and calculated mass values of putative archaeal lanthipeptides

BGC	Sequence	Name	Modification	Expected m/z $[M+2H]^{2+}$	Observed m/z $[M+2H]^{2+}$	△ppm	Peptide motif revealed by LC-MS/MS
alnα	GCGFTCSPFSSW	**1**	dehydration*2	621.7390	621.7382	1.2867	GFCDhaSPFCSW
		**1a**	dehydration*2<br>hydroxylation*1	629.7365	629.7350	2.3820	SPFSCS
alnβ	GLPSASMYSFEHCC	**2**	dehydration*2<br>methylation*1	755.3016	755.3012	0.5296	mGLP
		**2a**	dehydration*2	748.2938	748.2935	0.4009	P
		**2b**	dehydration*2<br>methylation*2	762.3095	762.3085	1.3118	mGLP
		**2c**	dehydration*2<br>methylation*1<br>hydroxylation*1	763.2991	763.2986	0.6551	mGLP
		**2d**	dehydration*2<br>methylation*2<br>hydroxylation*1	770.3069	770.3064	0.6491	mGLP

Supplementary Table 4. NMR data for archalan α (1) in d_6-DMSO (500 MHz ^{1}H NMR, 125 MHz ^{13}C NMR).

Residue	Position	δ_C	δ_H, mult	H-H COSY	HMBC
**Gly-1**	1	165.9, C			
	2	40.0, CH_2	3.58, dd	7.96	165.9, 169.6
	NH_2		7.96	3.58	165.9, 40.0
**Ala-2**	1	169.9, C			
	2	51.9, CH	4.73, td, *J* = 8.1, 4.8 Hz	2.78, 8.84	169.9, 165.9, 32.6
	3	32.6, CH_2	2.75, 2.81	4.73	51.9, 43.4
	NH		8.84, d, *J* = 8.4 Hz	4.73, 2.79	165.9, 51.9
**Gly-3**	1	167.9, C			
	2	43.1, CH_2	3.72, 3.63	8.62	168.0
	NH		8.62, dd, *J* = 8.1, 4.8 Hz	3.72	169.9
**Phe-4**	1	169.8-169.9, C			
	2	55.8, CH	4.28, td, *J* = 9.2, 5.8 Hz	2.81, 8.10	37.1, 169.9, 137.7
	3	37.1, CH2	2.81, 2.95	4.28	55.7, 128.8, 137.5, 169.8-169.9
	4	137.4, C			
	5/9	128.8, CH	7.27		137.5, 128.2, 128.8
	6/7/8	126.4, 128.2, CH	7.17-7.20		128.8, 126.2, 37.2
	NH		8.11, d	4.28	168.0, 55.7
**Abu-5**	1	169.4, C			
	2	56.2, CH	4.00, dd, *J* = 11.1, 7.8 Hz	3.06, 7.86	43.4, 169.4
	3	43.4, CH_2	3.07	1.24, 4.01	56.3, 32.6
	4	19.5, CH_3	1.24, d, *J* = 6.7 Hz	3.06	43.4, 56.2
	NH		7.86	4.01	169.8/9, 43.4
**Ala-6**	1	169.8-169.9, C			
	2	51.8, CH_2	4.61	8.70, 2.71	169.9, 170.1, 32.1
	3	32.1, CH_2	2.66, 2.73	4.61	33.0, 51.6, 169.8-169.9
	NH		8.70, d, *J* = 7.8 Hz	4.62	51.9, 169.4
**Ser-7**	1	169.9, C			
	2	54.7, CH_2	4.35, dt, *J* = 8.0, 5.5 Hz	8.16， 3.53	61.5, 169.9
	3	61.5, CH_2	3.53	4.35	
	NH		8.16, d, *J* = 8.1 Hz	4.34	169.9, 54.7
**Pro-8**	1	171.0, C			
	2	60.3, CH	4.62		20.8, 30.9, 45.9, 171.0
	3	30.9, CH_2	1.73, 1.45	4.64, 0.75	46.0, 20.9, 60.3,
	4	20.8, CH_2	1.43, 0.75	1.75	46.0, 30.8
	5	46.0, CH_2	3.14, 3.37	1.44	31.0
**Phe-9**	1	171.6, C			
	2	55.7, CH	4.62	2.95, 2.71, 7.94	37.3, 171.6
	3	37.3, CH_2	2.91, 3.07/2.78	4.61	55.7, 128.7, 137.5
	4	137.5, C			
	5/9	128.8, CH	7.27		137.5, 128.2, 128.8
	6/7/8	126.3, 128.2, CH	7.17-7.20		128.8, 126.2, 37.2
	NH		7.95	4.61	171.0
**Ala-10**	1	169.9, C			
	2	53.0, CH	4.50	8.09, 2.92,	32.8, 169.9
	3	32.8, CH_2	2.92, 2.79	4.50	55.9, 32.1, 55.8
	NH		8.09, d, *J* = 7.6 Hz	4.52	171.6, 32.8, 53.0
**Ser-11**	1	169.6, C			
	2	52.3, CH	4.48	7.85	62.2, 169.6
	3	62.2, CH_2	3.30		52.3, 169.6
	NH		7.85, t, *J* = 7.3 Hz	4.46	169.9

**Trp-12**	1	172.9, C			
	2	52.6, CH_2	4.47	3.59, 8.05	109.6, 172.9, 27.0
	3	27.0, CH_2	3.08, 3.20	4.46	109.6, 123.6, 127.1, 172.9
	4	109.4, C			
	5	123.6, CH	7.16	10.81	135.9, 109.4
	NH		10.82, s	7.16	135.9, 127.1, 123.6, 109.4
	7	135.9, C			
	8	111.2, CH	7.32, d, J = 8.1 Hz	7.06	118.3, 127.1
	9	120.7, CH	7.05, t, J = 7.52	7.32	118.3,135.9
	10	118.3, CH	6.96, t, J = 7.4 Hz	7.51	111.2, 127.1
	11	118.0, CH	7.52, d, J = 7.9 Hz	6.96	135.9, 120.8, 109.4
	12	127.1, C			
	NH		8.07, d, J = 7.6 Hz	4.46	169.6, 53.2, 27.1
	COOH		12.60, s		

Supplementary Table 5. Advanced Marfey's analysis of archalan α (Retention times (in min) constituent amino acids derivatized with D/L-FDLA).

Residue	L-FDLA	D-FDLA	Configuration
Serine	14.48	14.76	L
Proline	15.69	16.56	L
Phenylalanine	17.56	19.24	L
Methyllanthionine †	13.14, 13.61	12.74, 12.93	DL
Lanthionine	17.53	18.23	LL
Tryptophan *	ND	16.6	L

† Retention time consistency with the FDLA derivatives of Nisin's D, L-methyllanthionine.

* Detecting by HR-LCMS and comparing with standard L-Trp-L/D-FDLA, ND represents no data available for the low intensity.

Supplementary Table 6. Strains used in this study

Strain	Genome accession number	Source
Haloarcula argentinensis	GCF_000336895.1	CGMCC 1.6166
Haloferax larsenii	GCF_000336955.1	CGMCC 1.4296
Haloferax mediterranei	GCF_000306765.2	CGMCC 1.2087
Halomicrobium mukohataei	GCF_000023965.1	CGMCC 1.6192
Halorussus litoreus HD8-51	GCF_003382685.1	CGMCC 1.15333
Halorussus salinus YJ-37-H	GCF_004765815.2	CGMCC 1.12571
Haloferax volcanii	GCF_000025685.1	CGMCC 1.8356
Staphylococcus aureus	/	ATCC 25923
Bacillus subtilis 168	/	Our lab
Escherichia coli DH5α	/	Our lab

H. argentinensis and *H. mukohataei* isolated from the soils of the Argentine salt flats; *H. larsenii* from a mixture of mud and brine from a solar saltern in China; *Haloferax mediterranei* from Spanish saltern; *H. litoreus* HD8-51 from the salted brown alga *Laminaria*; *H. salinus* YJ-37-H from Yangjiang marine solar saltern in China; and *H. volcanii* from the Dead Sea.

References:

1. Castro I, Costa H, Turgeman-Grott I, Allers T, Mendo S, Caetano T. The lanthipeptide biosynthetic clusters of the domain Archaea. *Microbiol Res* **253**, 126884 (2021).

2. Tietz JI, *et al.* A new genome-mining tool redefines the lasso peptide biosynthetic landscape. *Nat Chem Biol* **13**, 470-478 (2017).
